# Conceptus Elongation, Implantation, and Early Placental Development in Species with Central Implantation: Pigs, Sheep, and Cows

**DOI:** 10.3390/biom15071037

**Published:** 2025-07-17

**Authors:** Gregory A. Johnson, Thainá Minela, Heewon Seo, Fuller W. Bazer, Robert C. Burghardt, Guoyao Wu, Ky G. Pohler, Claire Stenhouse, Joe W. Cain, Zachary K. Seekford, Dallas R. Soffa

**Affiliations:** 1 Department of Veterinary Integrative Biosciences, College of Veterinary Medicine and Biomedical Sciences, Texas A&M University, College Station, TX 77843, USA; 2 Department of Animal Science, College of Agriculture and Life Sciences, Texas A&M University, College Station, TX 77843, USA; 3 Department of Animal and Avian Sciences, College of Agriculture and Natural Resources, University of Maryland, College Park, MD 20742, USA; 4 Department of Animal Science, Pennsylvania State University, University Park, PA 16802, USA

**Keywords:** livestock, pigs, sheep, cows, pregnancy, conceptus, elongation, implantation, placentation

## Abstract

Species have different strategies for implantation and placentation. Much can be learned about general molecular and cellular biology through the examination and comparison of these differences. To varying degrees, implantation in all species includes alterations in epithelial polarity, the transformation of the endometrial stroma, the differentiation of the trophoblast, cell-to-cell and tissue-to-tissue signaling through hormones, cytokines, and extracellular vesicles, and the alteration of the maternal immune system. This review focuses on implantation in pigs, sheep, and cows. These species share with mice/rats and humans/primates the key events of early embryonic development, pregnancy recognition, and the establishment of functional placentation. However, there are differences between the pregnancies of livestock and other species that make livestock unique biomedical models for the study of pregnancy and cell biology in general. Pig, sheep, and cow conceptuses (embryo/fetus and associated placental membranes) elongate prior to implantation, displaying central implantation, extended periods of conceptus attachment to the uterus, and epitheliochorial (pigs) and synepitheliochorial (sheep and cows) placentation. This review will discuss what is understood about how the trophoblast and extraembryonic endoderm of pig, sheep, and cow conceptuses elongate, and how a major goal of current in vitro models is to achieve conceptus elongation. It will then examine the adhesion cascade for conceptus implantation that initiates early placental development in pigs, sheep, and cows. Finally, it will conclude with a brief overview of early placental development in pigs, sheep, and cows, with a listing of some important “omics” studies that have been published.

## 1. Introduction

Placentation in pigs, sheep, and cows is non-invasive. Therefore, there are more physical barriers to potentially limit the movement of nutrients and other products from the uterus to the embryo/fetus than are present in species that have invasive placentation. However, newborn pigs, sheep, and cows are relatively mature compared to the newborn of rodents and primates. Clearly there are fundamental differences in the strategies for implantation and placentation between pigs, sheep, and cows versus mice/rats and humans/primates. These differences make livestock unique biomedical models that can be, and have been, utilized for the scientific investigation of pregnancy and cell biology in general. First, the conceptuses (embryo/fetus and associated placental membranes) of pigs, sheep, and cows elongate prior to, and during, the initial stages of implantation, a process still not well understood mechanistically, but one that offers the potential for providing new molecular and cellular insights into how cells interact and work together to proliferate, migrate, and change morphology within a somewhat hypoxic environment. Second, the conceptuses of pigs, sheep, and cows are not implanted into the endometrial wall. Rather, they attach to the surface epithelium of the endometrium, and pregnancy develops within the lumen of the uterine horn. The blastocysts of all eutherian species, including the biomedically significant rodent and primate research animal models, and humans, initially attach to the surface of the endometrium, but in most cases, attachment is quite brief followed, by the breaching of the endometrial luminal epithelium (LE) and invasion into the underlying stroma. In contrast, the attachment of the conceptus to the endometrial LE in livestock is a prolonged event. Some investigators consider attachment for implantation to span from day 13 to day 25 of gestation in pigs, offering an excellent model for the study of this aspect of implantation that is more difficult to examine in rodent and primate animal models. And third, mature placentation is epitheliochorial in pigs, and synepitheliochorial in sheep and cows. Therefore, pigs, sheep, and cows offer the opportunity to compare and contrast implantation in a way that utilizes minor changes in trophoblast differentiation and endometrial remodeling in the case of pigs. Implantation utilizes moderate trophoblast differentiation and endometrial remodeling in the case of sheep and cows and utilizes more extensive trophoblast differentiation and endometrial remodeling in the case of species with hemochorial placentation, including rodents and humans. Much can be learned about general molecular and cellular biology through the examination and comparison of these differences. To varying degrees, implantation in all species includes alterations in epithelial polarity, the transformation of the endometrial stroma, the differentiation of the trophoblast, cell-to-cell and tissue-to-tissue signaling through hormones, cytokines, and extracellular vesicles, and the alteration of the maternal immune system. Finally, although somewhat simplistic in structure, with pigs having epitheliochorial and diffuse placentation and cows and sheep having synepitheliochorial and cotyledonary placentation, placentation in these species is not primitive, and has evolved from more invasive placental types including hemochorial, endotheliochorial, and discoid [1]. Listed here are a few citations for reviews that readers may find valuable for the study of pregnancy in the livestock species. Although not exhaustive, the list is a start [2,3,4,5,6,7,8,9,10,11,12,13,14]. Other relevant reviews can be found in the following text.

## 2. The Trophoblast and Extraembryonic Endoderm of Pig, Sheep, and Cow Conceptuses Elongate Prior to, and During, Implantation

**Figure 1:**
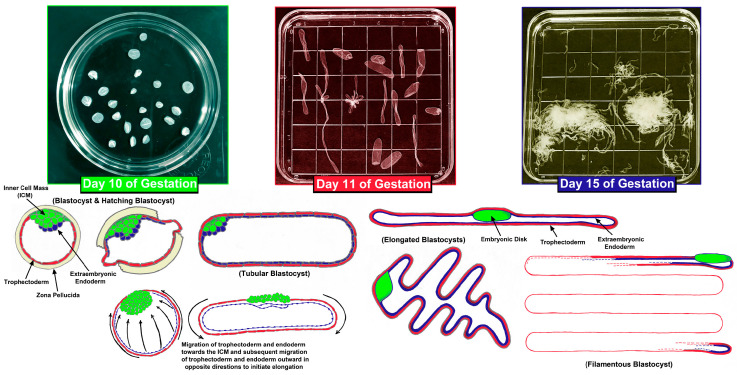
Conceptus elongation in the pig. The top panels are Petri dishes in which uterine flushings from days 10, 11, and 15 of gestation contain conceptuses undergoing rapid morphological changes. The bottom panels graphically illustrate the migration of trophoblast and extraembryonic endoderm cells during conceptus elongation between days 10 and 15 of gestation. Electron micrographs suggest that the trophoblast and endoderm migrate towards the inner cell mass and then subsequently migrate outward in opposite directions to initiate elongation in pigs. Although pig conceptuses elongate to a greater extent than the conceptuses of sheep and cows, the process is believed to be mechanistically similar among the three species. For the conceptus in the bottom right corner the extraembryonic endoderm extends across the entire length of the trophoblast and the trophoblast layer is continuous, similar to the adjacent conceptuses. Both have been drafted in an abbreviated manner to simplify the drawing.

The changes in morphology of elongating pig, sheep, and cow conceptuses have been well described [6,8,9,10,15,16,17,18,19,20,21,22,23,24,25,26,27,28]. After fertilization within the oviduct, the zygote undergoes cleavage divisions which result in a mass of cells called the morula that remains encased within the zona pellucida of the ovulated egg. Embryos enter the uterus at the 4- to 8-cell stage in pigs where the morula then rapidly forms, but the morula that enters the uterus around day 6 post-fertilization in sheep and between days 4 and 6 in cows. Blastocysts form when early embryonic cells differentiate into the inner cell mass (ICM), trophoblast, and extra-embryonic endoderm with the latter surrounding a round cavity termed the blastocoel. The ICM will eventually become the embryo/fetus proper (primitive ectoderm, mesoderm, and endoderm) as well as give rise to a subjacent layer of extraembryonic cells, the trophoblast will eventually become the placental chorion, and the extraembryonic endoderm differentiates into the visceral endoderm and parietal endoderm of the yolk sac. These tissues are now collectively termed the conceptus. Conceptuses ‘hatch’ from the zona pellucida on about day 7 in pigs, days 6 to 7 in sheep, and days 9 to 10 in cows. At hatching, pig conceptuses are 0.5 to 1 mm diameter spheres that increase in size to 2 to 6 mm spheres by day 10 of gestation (Figure 1). In the day 10 blastocysts of pigs an elongation zone of densely packed endoderm and trophoblast cells extends from the ICM to the tip of the blastocyst. Then, through alterations in microfilaments and junctional complexes of trophoblast cells and formation of filopodia by endodermal cells, there is cell migration outward in opposite directions to morphologically transition to large 15 mm spheres and then to 50 mm tubes. Subsequent elongation then advances at a rate of 30 to 45 mm/h during the transition from tubular to filamentous forms, primarily by proliferation, morphological remodeling, and migration of the trophoblast and endoderm to reach the dimensions of 1 mm by 100 to 200 mm by day 11 (Figure 1). During subsequent conceptus growth there is further rapid elongation of the 100 to 200 mm long conceptus to a conceptus of 800 to 1000 mm in length by day 15 of pregnancy (Figure 1). Cow blastocysts are 2 mm spheres on day 13, elongate to 60 mm by day 16, and reach 200 mm by day 19 of gestation, whereas, sheep blastocysts are 0.14 mm spheres on day 4, 0.4 mm spheres by day 10, elongate to 33 mm filaments by day 12, and are 150 to 190 mm by day 15, extending through the uterine body into the contralateral uterine horn by days 16 to 17 of pregnancy.

**Figure 2:**
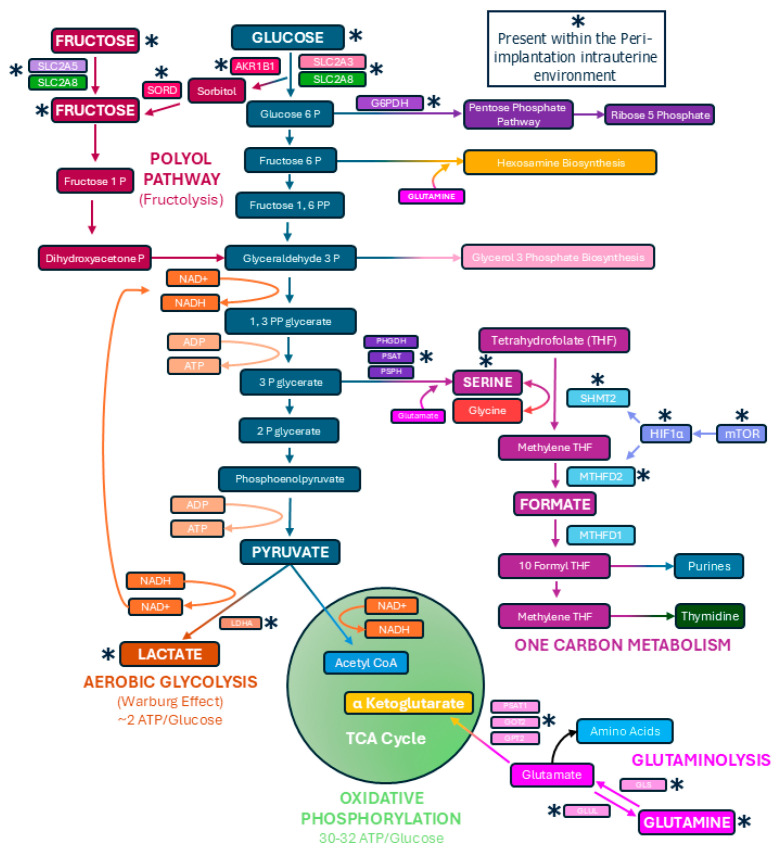
Pathways utilized by elongating conceptuses of pigs and sheep to metabolize the hexose sugars glucose and fructose. Shown are metabolic pathways confirmed to be active within intrauterine environment of pigs. Although active glutaminolysis pathway has yet to be established in sheep conceptuses, elongating sheep conceptuses are active in one-carbon metabolism, the pentose phosphate pathway, the polyol pathway (fructolysis), and the conversion of pyruvate into lactate. There is also evidence for hexosamine biosynthesis in pig and sheep conceptus trophoblasts.

The elongation of pig, sheep, and cow conceptuses requires extensive trophoblast and extraembryonic endoderm cell proliferation, migration, and morphological remodeling. Pig and sheep conceptuses support these processes through ATP supplied, at least in part, by the hexose sugars glucose and fructose [29,30,31,32,33]. Indeed, glucose and fructose increase in the uterine lumen of pigs and sheep when conceptuses elongate [34,35,36]. Glucose and fructose are transported across cell membranes by the facilitated diffusion transporters of the solute carrier 2A (SLC2A) family. SLC2A1 is responsible for the basal uptake of glucose, while SCL2A3 has the capacity to transport glucose at much higher levels [37]. SLC2A5 transports fructose, but not glucose, and SLC2A8 transports both glucose and fructose into and out of cells [38]. Pig conceptus trophoblast and later chorionic epithelium express SLC2A1, SLC2A3, SLC2A5, and SLC2A8 (Figure 2). The levels of SLC2A3 are higher than SLC2A1 during the peri-implantation period, and SLC2A3 replaces the expression of SLC2A1 in the tall columnar cells at the tops of uterine–placental folds and in the chorionic epithelium of areolae during later stages of gestation. SLC2A5 mRNA increases in the chorionic epithelium as pregnancy progresses, while SLC2A8 protein is abundant at both the apical and basal surfaces of the trophoblast of elongating and implanting conceptuses. Later in gestation, SLC2A8 expression is limited to the chorionic epithelium at the tops of uterine–placental folds and areolae [36,39]. The trophoblast cells of sheep conceptuses express both SLC2A1 and SLC2A3 [40]. SLC2A3 is more abundant than SLC2A1. SLC2A3 is present at the apical surface, but SLC2A1 is localized to the basal surface of the trophoblast cells of sheep [40,41].

Conceptuses consume available oxygen and nutrients to support elongation, and this results in a hypoxic intrauterine environment in which elongating and implanting conceptuses are under metabolic stress [42,43]. The metabolism of the hexose sugars primarily occurs through the tricarboxylic acid (TCA) cycle and oxidative phosphorylation, which is an efficient way to produce ATPs [44]. However, cells that undergo high levels of proliferation and migration, including cancer cells and activated lymphocytes, primarily perform glycolysis, even in the presence of sufficient oxygen. This is known as aerobic glycolysis or the Warburg effect [44,45,46]. Aerobic glycolysis to form lactate is a relatively inefficient means to produce ATP, but the glycolytic intermediates that accumulate can be funneled into the pentose phosphate pathway, hexosamine biosynthesis, and one-carbon metabolism for the synthesis of nucleotides and active phosphorylation of enzymes needed for cell division [44,45,46]. In response to a hypoxic environment, the elongating conceptuses of pigs, sheep, and cows likely switch from oxidative to glycolytic metabolism in a manner similar to that of tumors and activated immune cells that remain highly active within hypoxic environments (Figure 1) [30,31,32,33]. Indeed, when the elongating conceptuses of pigs and sheep were incubated with glucose and/or fructose they generated significant amounts of lactate indicating they are capable of active aerobic glycolysis [33,47,48,49,50].

Proliferating cells often synthesize serine at greater levels than resting cells, and recent studies have demonstrated that pig and sheep conceptuses utilize one-carbon metabolism and the pentose phosphate pathway during elongation (Figure 2) [30,32,33]. The reactions of serineneogenesis are catalyzed by the successive actions of the enzymes phosphoglyceride dehydrogenase (PHGDH) which converts 3-phosphoglycerate (3PG) to 3-phosphohydoypyruvate (PHP), phosphoserine aminotransferase 1 (PSAT1) which converts PHP to 3-phosphoserine, and phosphoserine phosphatase (PSPH) which converts 3-phosphoserine to serine, with glutamate providing the amino group for this reaction (Figure 2). Serine enters mitochondria for incorporation into one-carbon metabolism in which serine hydroxymethyltransferase 2 (SHMT2) catalyzes the conversion of serine and tetrahydrofolate (THF) to glycine and 5,10-methylene tetrahydrofolate (mTHF). mTHF is then converted to formate through the actions of methylenetetrahydrofolate dehydrogenase 2 (MTHFD2). Formate is transported to the cytoplasm for the generation of purines required for RNA and DNA synthesis (Figure 2). In pigs, the endometrial LE expresses PHGDH, PSAT, and PSPH to convert 3PG and glutamate to serine, and this serine is secreted into the uterine lumen for exposure to elongating conceptuses in which the mechanistic target of rapamycin (mTOR) pathway has been activated in response to the hypoxic intrauterine environment. mTOR increases the expression of hypoxia inducible factor 1 alpha (HIF1α) which then drives increased expression of SHMT2 and MTHFD2 and the production of formate by the elongating conceptuses (Figure 2) [31,33,49,51]. Therefore, the endometrial LE produces and releases serine that is converted to purines and 2-deoxy-thymidilate in conceptus trophoblast via activation of one-carbon metabolism by the mTOR-HIF1α pathway (Figure 2). In sheep mRNAs for PHGDH, PSAT1, PSPH, SHMT1, SHMT2, MTHFD1, MTHFD2, MTHFD1L, and MTHFD2L are present in elongating conceptuses, and incubation of sheep conceptuses with glucose or fructose results in the formation of formate [50,51,52]. Further, the inhibition of SHMT2 mRNA translation increased conceptus mortality in sheep [53]. Therefore, both one-carbon metabolism and serinogenesis are active metabolic pathways in elongating sheep conceptuses, and hexose sugars are substrates for generating formate required for nucleotide synthesis [52].

Aerobic glycolysis also provides biosynthetic intermediates for the synthesis of ribose. Glucose-6-phosphate dehydrogenase (G6PDH) drives the conversion of glucose-6-phosphate for entry into the pentose phosphate pathway and eventual production of ribose-5-phosphate. The conceptus trophoblast of pigs expresses G6PDH, and when elongating pig conceptuses were incubated with glucose or fructose, glucose, but not fructose, contributed carbons into the pentose phosphate pathway (Figure 2) [33]. Similarly, elongating sheep conceptuses preferentially oxidized glucose over fructose when they were present at equal concentrations (e.g., 4 mM), but both glucose and fructose contributed carbons to the pentose phosphate pathway and the physiological concentrations of fructose in the allantoic fluid of ruminants and swine can be 30-times greater than those of glucose (e.g., ~30 mM fructose vs. ~1 mM glucose) [29,47].

If elongating pig and sheep conceptuses direct the majority of carbons generated from the hexose sugars away from the TCA cycle, and use carbons for aerobic glycolysis, one-carbon metabolism, and the pentose phosphate pathway, this limits available pyruvate to maintain the TCA cycle within mitochondria. Cancer cells convert glutamine into TCA cycle intermediates to maintain the TCA cycle in a process called anaplerosis [54,55]. Glutamine is converted into glutamate via glutaminase (GLS), and then glutamate is converted into α-ketoglutarate (α-KG) through the actions of glutamate dehydrogenase (GLUD) or the aminotransferases glutamate–oxaloacetate transaminase (GOT), glutamate–pyruvate transaminase (GPT), and phosphoserine transaminase (PSAT). As a component of the TCA cycle, α-KG is a substrate that maintains active oxidative phosphorylation and production of the 30 to 32 ATPs generated from each molecule of glucose. Glutamine increases in the uterine lumen of pregnant pigs during the peri-implantation period, and pig trophoblast cells proliferate in response to glutamine in the culture medium [56]. Indeed, pig conceptus expression of GLS and GLUL increases during the peri-implantation period, with GLS protein localized to the conceptus trophoblast on Day 15 while GLUL protein is localized to the conceptus endoderm on day 13 of gestation, suggesting that conceptus endoderm synthesizes glutamine, and conceptus trophoblast then consumes glutamine for glutaminolysis during elongation (Figure 2) [31]. Such an exquisite inter-cellular cooperation ensures the net formation of glutamine for use by trophoblasts. In addition, PSAT1 protein is highly abundant in conceptus trophoblast cells, and mRNAs for PSAT1, GOT2, and GPT2 are present in conceptuses on day 15 of the peri-implantation period, whereas GLS2 and GLUD1/2 are not detectable in those same cells (Figure 2). Therefore, elongating porcine conceptuses have the potential to perform glutaminolysis through the GLS-PSAT1 metabolic pathway (Figure 2) [31,33].

## 3. A Major Goal of Current In Vitro Models Is to Achieve Conceptus Elongation

Our understanding about how pig, sheep, and cow conceptuses elongate at a mechanistic level has been limited somewhat by the fact that conceptus elongation has not been achieved in vitro. During conceptus elongation and implantation there is dynamic but controlled tissue remodeling. Current in vitro models lack many physiochemical and physiomechanical characteristics present in the tissues of intact implantation sites including molecules and that initiate cell-to-cell signaling, both paracrine and mechanical in nature. In vivo, the process of implantation does not occur in isolation. In pigs, sheep, and cows the elongating conceptus interacts with an endometrium that is developing receptivity to eventual implantation, with the conceptuses and endometrium of each species anticipating different, if modest, levels of invasiveness into the uterine wall [57,58,59,60]. Implantation initiates the establishment of what might be termed a ‘functional syncytium’, where various types of cells from divergent tissues, the uterus and placenta, function synergistically to sustain pregnancy. Due to the inherent complexity, these processes have not been replicated in vitro. However intense efforts to achieve conceptus elongation in vitro are currently being pursued.

Models for pre-hatching embryonic development are being refined to mimic physiological conditions. Incorporating cell movement [61,62], co-culture with organoids [63], or inclusion of microfluidics [64] can improve development rates and embryonic quality up to the blastocyst stage. The elongation of the conceptus commences following the rupture of the zona pellucida [65]. The trophoblast is no longer constrained within the zona and this allows the physical freedom to undergo the intense trophoblast and extraembryonic endoderm cell proliferation, migration, and changes in morphology that underlie conceptus elongation [66,67]. These processes require mechanosensation and mechanotransductional support from the uterus (mechanical forces exerted on the conceptus) [68,69] and access to growth factors and nutrients present in the histotroph [70,71]. Attempts have been made to encapsulate pig and cow blastocysts in alginate hydrogels droplets [72,73,74] and to culture cow blastocysts in glass tunnels coated in agarose gel [75,76] to provide a structural framework for conceptus elongation outside of the uterine lumen. It is encouraging that these models support morphological changes leading to increased length of the trophoblast, but enthusiasm is tempered because true elongation does not occur, and the ICM presents extensive necrotic areas and abnormal morphology [75]. Developing 3D in vitro models, or tissue engineering, will likely be necessary to achieve elongation in vitro. Dynamic systems are being developed that combine cells, connective tissue scaffolds or commercial extracellular matrix derivates, and circulating biomolecules via microfluidics in what can be considered engineered organs [69,77,78,79,80]. Ultimately, the goal is for these systems to emulate the function, physical structure, and regulatory mechanisms of organs. In cattle, an oviduct-on-a-chip was developed to aid in early embryonic development and further characterize maternal-embryonic interactions [81,82]. Recently, fragments of endometrial GE were cultured in 2D and 3D systems. The in vivo phenotype as well as the tissue architecture were maintained in this 3D culture system [83]. Responsiveness to progesterone and production of classical endometrial GE proteins, including SERPINA14, were observed when co-cultured with stromal cells [84], reiterating the necessity of multiple cell models to achieve proper tissue function.

**Figure 3:**
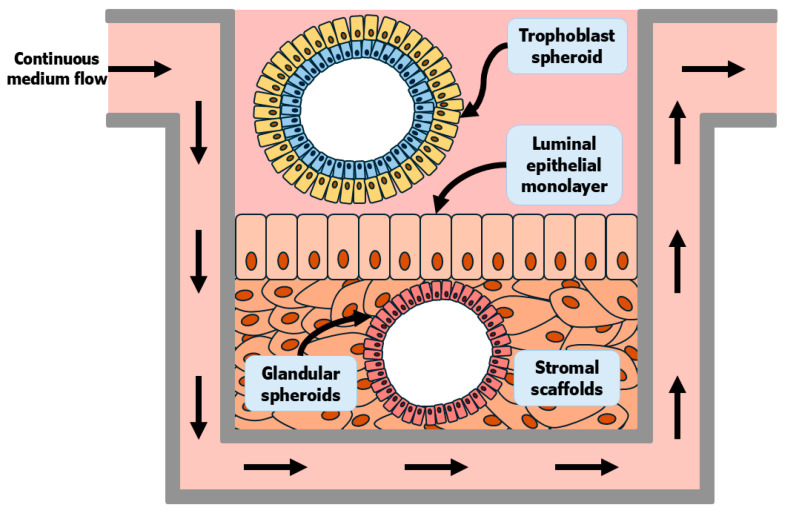
Adapted from Tinning and collaborators (2023) [67]. Conceptus elongation is still not well understood in mechanistic detail because elongation has not been reproduced in vitro. This is an area of current intense interest for the field of early pregnancy in the livestock species. Shown is a recreation of a conceptualized macrofluidic system that could emulate local and systemic processes that occur during pregnancy. This system would be nourished via continuous culture media flow. The architecture of the tissue should be sustained by stromal scaffolds containing cultured endometrial spheroids. The apical surface would be populated with an endometrial LE monolayer. Above the endometrial LE, either trophoblast blastoids or trophoblast spheroids would be cultured in appropriate media, while still being exposed to products from the tissue cultured within the same well.

One recent advancement was the development of human endometrial “assembloids” (epithelial organoids + dense stromal cells + self-made endothelial bed) cultured in collagen-based media and with an exposed apical surface [85]. These structures were responsive to steroid hormone stimulation and presented comparable molecular signatures and architecture to the in vivo endometrium. Within 3 days of co-culture, blastoids derived from embryonic stem cells had established apposition, adhesion, and invasion into the assembloid stromal scaffold [85]. In cattle, co-culture of trophoblast spheroids and endometrial epithelial cells was proposed as a model to study the mechanisms of conceptus attachment [86]. When co-cultured with endometrial epithelial cells, the spheroids had greater expression of classical markers of cell-to-cell adhesion, such as beta 3 (β3) integrin subunit (ITGB3), as well as N-cadherin, which is also expressed in actively attaching conceptuses on day 22 of pregnancy [87]. Tinning and collaborators (2023) [67] have conceptualized a macrofluidic system that could emulate local and systemic processes that occur during pregnancy (Figure 3). This system would be nourished via continuous culture media flow. The architecture of the tissue should be sustained by stromal scaffolds containing cultured endometrial spheroids. The apical surface would be populated with an endometrial LE monolayer. Above the endometrial LR, either trophoblast blastoids or trophoblast spheroids would be cultured in appropriate media, while still exposed to products from the tissue cultured within the same well [67].

## 4. The Adhesion Cascade for Conceptus Implantation That Initiates Early Placental Development in Pigs, Sheep, and Cows

**Figure 4:**
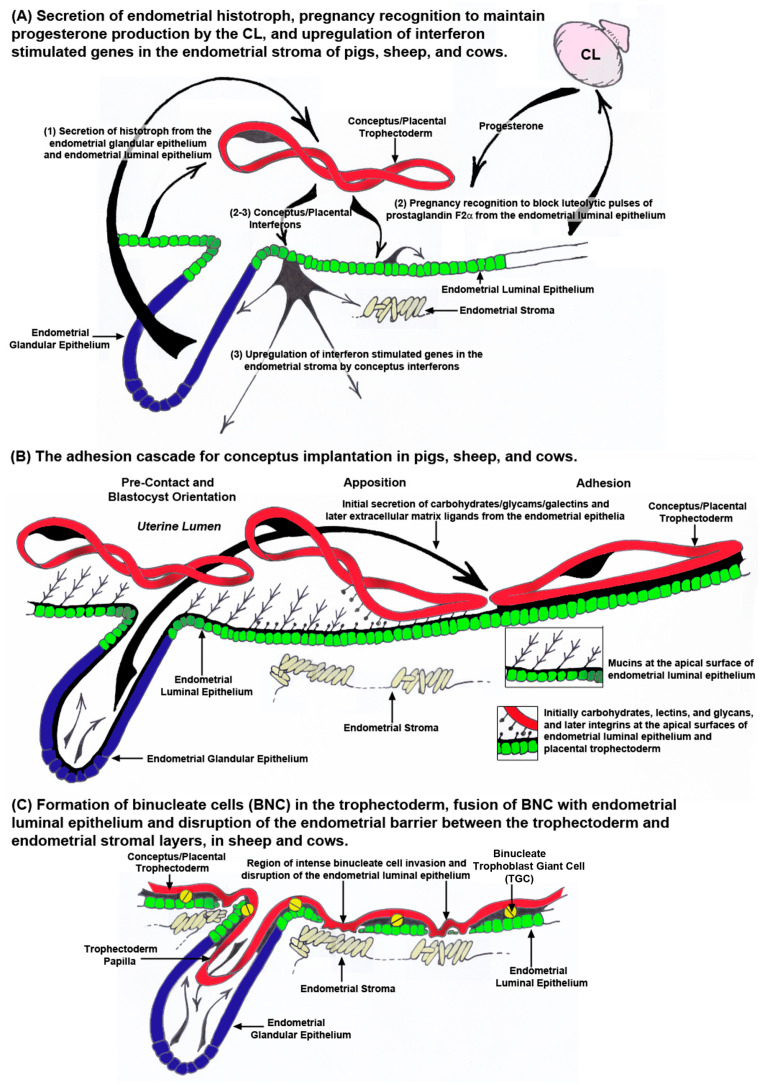
The peri-implantation period of pregnancy for pigs, sheep, and cows. Panel A illustrates pre-implantation events. (1) Histotroph is secreted from the endometrial luminal epithelium (LE) and glandular epithelium (GE) of pigs and GE of sheep and cows in response to progesterone and other factors. When conceptuses have elongated sufficiently, they secrete pregnancy recognition factors, primarily estrogen in the pig and interferon tau (IFNT) in sheep and cows. In addition, the conceptus IFNs, IFN gamma (IFNG) in the pig and IFNT in sheep and cows upregulate the expression of interferon stimulated genes (ISGs) in the endometrial stroma. Panel B illustrates the adhesion cascade for implantation. The elongating conceptuses of pigs, sheep, and cows orient the apical surface of the trophoblast to the apical surface of the endometrial LE. Removal of anti-adhesive mucins from the apical surface of the endometrial LE allows for close apposition and attachment of conceptus trophoblast. Initial juxtracrine interactions between the apical surface of the trophoblast and endometrial LE are mediated through carbohydrates, glycams, and galectins, supplied as histotroph, binding to carbohydrates, lectins, and glycans, present on the cell surfaces. Firm adhesion is later mediated through extracellular matrix (ECM) ligands, supplied as histotroph, binding to integrins on the cell surfaces. Pigs have true epitheliochorial placentation as the trophoblast does not breach the endometrial LE barrier to the underlying endometrial stroma. Panel C illustrates early stages of placentation in sheep and cows. The conceptuses of sheep and cows extend trophoblast papillae into the mouths of the endometrial GE perhaps for increased access to histotroph secreted by the GE. After adhesion, binucleate trophoblast giant cells (TGCs) begin to form and fuse with endometrial LE cells. These events result in significant disruption of the endometrial LE barrier to trophoblast interaction with the endometrial stroma.

Pigs, sheep, and cows undergo estrous cycles that include proestrus in which waves of follicular growth result in the development of a dominant follicle(s) and their secretion of estrogen, increases in the pituitary hormones luteinizing hormone (LH) and follicle stimulating hormone (FSH), estrus in which surges of estrogen and LH result in estrus behavior in the female preceding or near the time of ovulation, metestrus when estrogen and LH levels are low as luteinization of the ovarian follicle forms the corpus luteum/lutea (CL) for production of progesterone, and diestrus when the high levels of progesterone decrease precipitously in response to prostaglandin F2 alpha (PGF2α) from the uterine endometrium causing luteolysis of the CL(s) [88]. When the conceptuses of pigs, sheep, and cows elongate, they secrete proteins and steroids that block luteolysis of CL resulting in maintenance of high levels of progesterone necessary to support pregnancy, an event termed pregnancy recognition. Amongst these factors are type 1 interferon tau (IFNT) in sheep and cows, and estrogen and the type 2 IFN gamma (IFNG) in pigs (Figure 4A) [7]. Pig conceptuses secrete both type 1 IFN delta (IFND) and IFNG [89,90,91]. These IFNs are not considered to be antiluteolytic [92,93], but recent studies in which the synthesis of select factors in conceptuses was ablated utilizing CRISPR/Cas9 genome editing technology suggest that maintenance of early pregnancy in pigs is a servomechanism that requires the sequential effects of progesterone, estrogen, interleukin 1beta (IL1B), PGE2, and IFNG [94,95]. IL1B is required for sufficient elongation to allow secretion of estrogen as the pregnancy recognition signal, and later PGE2 and IFNG are required for the endometrial remodeling to support successful implantation and early placental development. In pigs, IFNG is delivered to the endometrial LE, at least in part, through extracellular vesicles [96], and IFNG alters populations of T cells within the endometrium as well as upregulates or induces multiple IFN stimulated genes (ISGs) in the endometrial stroma, events essential to maintain pregnancy through implantation (Figure 4A) [91,96,97,98,99,100,101]. The elongating conceptuses of sheep and cows secrete IFNT as the signal for maternal recognition of pregnancy, and IFNT also induces the expression of ISGs in the endometrial stroma [7,102,103,104,105]. In sheep, IFNT is delivered to the endometrial LE perhaps in part through extracellular vesicles [106,107] where it acts in a paracrine, antiluteolytic manner on the endometrial LE and superficial GE (sGE) to inhibit transcription of the estrogen receptor alpha (ESR1) gene. This precludes estrogen from inducing expression of oxytocin receptors (OXTR) through GC-rich SP1 promotor elements in the endometrial LE, thereby preventing oxytocin from inducing release of luteolytic pulses of PGF2α. The result is maintenance of the CL, the source of progesterone which is required to produce histotroph for a successful pregnancy (Figure 4A) [108,109,110,111].

**Figure 5:**
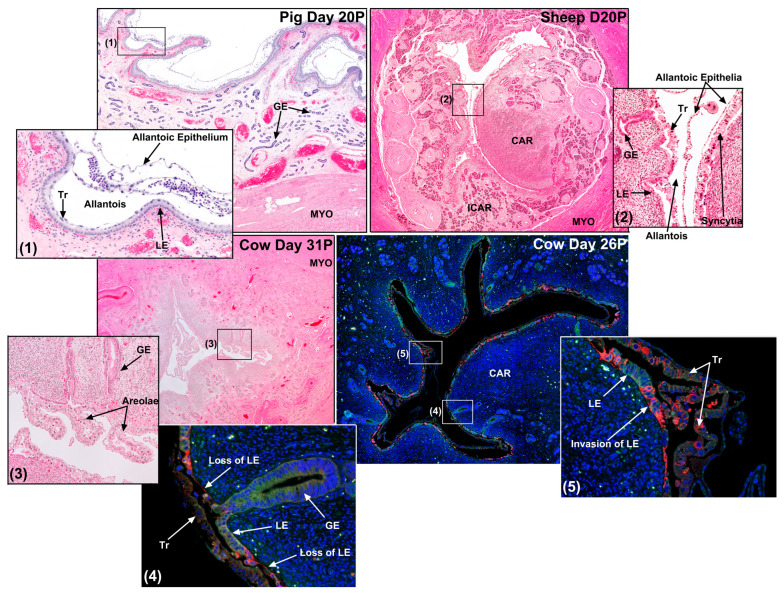
Sites of conceptus implantation in a pig, sheep, and two cows. Shown are implantation sites from a day 20 pregnant (20P; H&E staining) pig, a day 20P sheep (H&E staining), a day 31P cow (H&E staining), and a day 26P cow (green = immunofluorescence staining for epithelial-cadherin (E-cadherin); red = immunofluorescence staining for pregnancy-associated glycoproteins (PAGs); blue = DAPI staining of nuclei for histological reference). True epitheliochorial placentation is illustrated for the pig. A syncytium forms at the uterine–placental interface of the sheep. The loss of regions of the endometrial luminal epithelium (LE) is observed in the D26P cow. GE, endometrial glandular epithelium; CAR, endometrial caruncle; ICAR, intercaruncular endometrium; Tr, trophoblast; MYO, myometrium.

**Figure 6:**
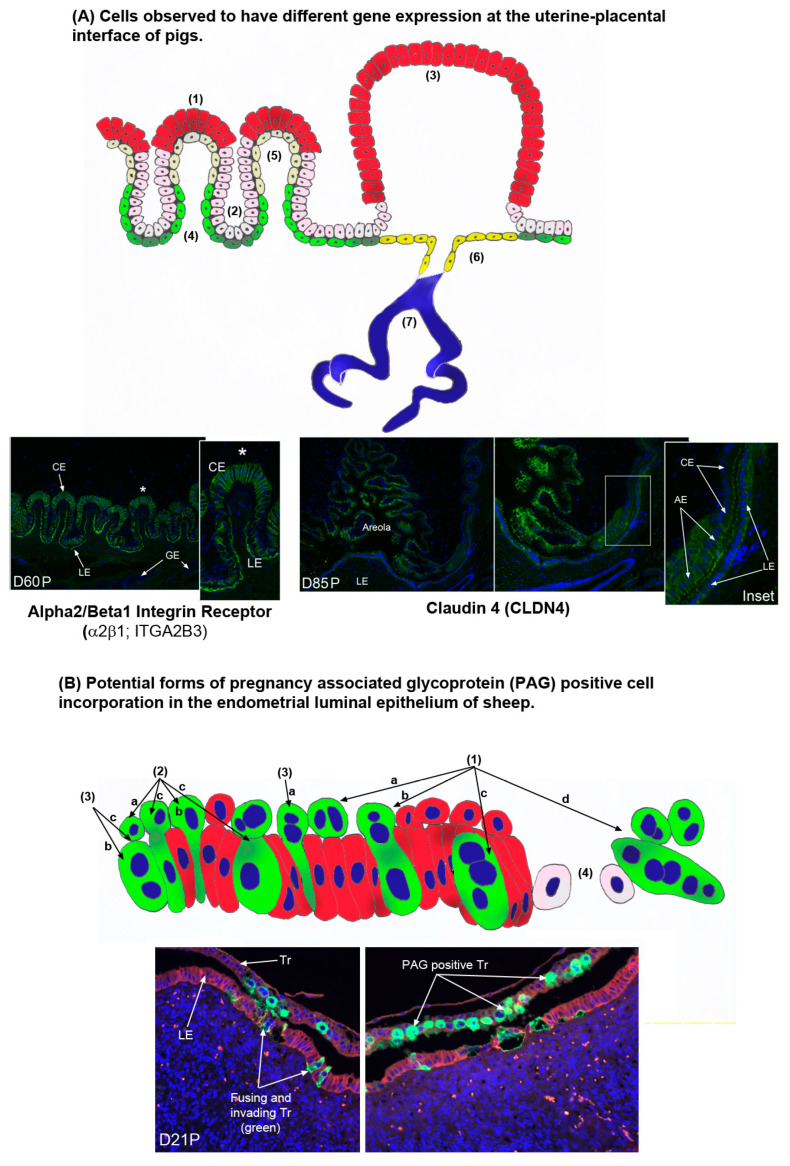
(**A**) There are multiple cell types at the uterine–placental interface of pigs that express genes differentially. These include the tall columnar chorion cells at the top of the uterine–placental folds indicated by the asterisks (1). Proteins expressed exclusively within these cells of the folds include solute carrier family 2A8 (SLC2A8) [39], glutaminase (GLS) [31], and brain-type creatine kinase (CKB) [123]. (2) The low cuboidal chorion cells at the bottom of the uterine–placental folds. A protein expressed exclusively within these folds cells is glutamine synthetase (GLUL) [31]. (3) The tall columnar chorion cells of the areolae. Amongst others, proteins and genes expressed in these cells, but not the chorion cells of the uterine–placental folds include cathepsin L (CTSL1) [124], SLC2A8 protein [39], mRNA for SLC2A3 [36], aquaporin 5 (AQP5) protein [123], the beta 3 (β3) integrin subunit (ITGB3) protein [125], and claudin 4 (CLDN4) protein as illustrated in the section from day 84 of pregnancy (D84P) in the figure. (4) The low cuboidal endometrial luminal epithelial (LE) cells at the bottoms of the folds of the uterine–placental interface. The integrin receptor α2β1 (ITGA2B1) protein is shown to be expressed by these cells in the section from day 60P in the figure. (5) The squamous endometrial LE cells at the tops of the folds of the uterine–placental interface. SLC2A3 mRNA is expressed exclusively within these fold cells [36]. (6) The endometrial LE cells lining the surface of areolae near the openings of the endometrial glandular epithelium (GE). OPN mRNA and protein are expressed in all endometrial LE cells except for these cells [125]. (7) Endometrial GE. Many proteins are expressed by the endometrial GE, but acid phosphate 5, tartrate resistant (ACP5, commonly referred to as uteroferrin) is only expressed in this cell type within the uterine environment of pregnant pigs where it is secreted and delivered to areolae as a component of histotroph [126,127]. (**B**) There may be multiple ways that pregnancy-associated glycoprotein (PAG)-positive cells are incorporated into endometrial luminal epithelium (LE) during syncytialization in sheep [9,57,59]. (1) Illustrates the formation of PAG positive binucleate trophoblast giant cells (TGCs) in the trophoblast (1a), fusion with an endometrial LE cell (1b), formation of a trinucleate syncytial cell composed of trophoblast and endometrial LE (1c), and continued fusion of TGCs with growing syncytia to form syncytial plaques at the uterine–placental interface of sheep (1d). However, PAG positive cells have been detected at the uterine–placental interface that are not predicted by these events. (2) Mononucleate PAG positive cells that are common in the trophoblast layer (2a) and sometimes appear to be invading between endometrial LE cells (2b), and mononucleate PAG positive cells are present in the endometrial LE layer allowing for the possibility that PAG positive TGCs may fuse with PAG positive endometrial LE cells, and PAG positive mononucleate Tr cells may fuse with PAG positive endometrial cells (2c). (3) PAG positive TGCs sometimes appear to be invading between endometrial LE cells and not fusing with LE cells (3a) and they are observed within the endometrial LE layer (3b), allowing for another possible means of forming PAG positive trinucleate cells, i.e., mononucleated trophoblast cells fusing with TGCs within the endometrial LE (3c). (4) Finally, fusion and invasion of PAG positive cells into the endometrial LE can lead to disruption and destabilization of the endometrial LE barrier to trophoblast contact with endometrial stroma. Bottom panels are uterine–placental interface from sheep that have been immunofluorescence stained for PAGs (green) and E-cadherin (red).

The term “implantation” is used to describe the processes of the attachment and invasion of the blastocyst into the endometrium of eutherian mammals. Implantation is a troublesome scientific term regarding pregnancy because it implies embedding of the blastocyst into the wall of the endometrium. Implantation is an appropriate term to use for humans, mice, and rats in which the trophoblast cells of the blastocysts breech the endometrial LE and invade into the endometrial stroma to provide a nest within the endometrium in which the blastocyst further develops. However, pigs, sheep, and cows exhibit central implantation, in which the blastocyst does not embed into the wall of the endometrium and instead develops within a placenta that is attached to the surface of the endometrium within the uterine lumen [8,9,112]. In pigs, sheep, and cows the placental trophoblast cells either do not invade into the endometrial LE, as is the case for pigs (Figure 4B, Figure 5 and Figure 6A), or there is limited invasion of trophoblast cells into the endometrial LE and fusion of trophoblast cells with endometrial LE to form syncytial cells or plaques, as is the case for sheep and cows, respectively (Figure 4C, Figure 5 and Figure 6B) [6,8,9,12]. Never-the-less all these species share the “Adhesion Cascade for Implantation”. The phases of this adhesion cascade include (1) shedding of the zona pellucida, (2) elongation of the trophoblast and extraembryonic endoderm in pigs, sheep, and cattle, but not humans, mice, and rats, (3) pre-contact and orientation of placental trophoblast to the endometrial LE, (4) apposition of trophoblast to endometrial LE, and (5) adhesion of the apical surface of the trophoblast to the apical surface of the endometrial LE (Figure 4B). Attachment of trophoblast to the endometrial LE first requires the removal of mucins, primarily mucin 1 (MUC1) from the glycocalyx of the endometrial LE that sterically inhibits adhesion. Mucin removal allows for direct physical interactions between a mosaic of carbohydrates, glycans (the O-glyclosidic linked carbohydrate portions of glycoconjucates), and lectins at the apical surfaces of the opposing trophoblast and endometrial LE which contribute to initial attachment of the trophoblast to the endometrial LE. These low affinity contacts are then strengthened by a repertoire of adhesions between membrane-bound integrins and their extracellular matrix (ECM) ligands which provide stable adhesion of trophoblast to endometrial LE for implantation (Figure 4B) [113,114,115,116,117,118,119,120]. Implantation is the beginning of placentation, and placentation differs significantly among species [121,122].

## 5. Conceptus Implantation in the Pig

Figure 5 shows a site of conceptus implantation in the pig from day 20 of pregnancy. What is understood about the “Adhesion Cascade for Implantation” in pigs has been reviewed in detail [3,4,8,9,120]. The amount of time required for the trophoblast of the pig conceptus to establish a firm enough attachment to the endometrial LE to support subsequent tissue remodeling to fold the uterine–placental interface is reported slightly differently by various investigators; however, it is reasonable to define this as day 13 to day 26 of gestation. By day 26, placentation initiates by developing uterine–placental folds that increase the surface area of the uterine–placental interface, and through the differentiation of cells that show cell-type specific expression of enzymes, receptors and transporters necessary to optimize transport of water, ions, and nutrients across the uterine–placental interface to support fetal growth and development (Figure 6A) [31,36,39,58,123,124,125,126,127,128,129,130]. The electron micrographic studies of Dantzer (1985) provide an excellent overview of the physical interactions between the conceptus and endometrium during this period [131]. Both the trophoblast of the conceptus and endometrial LE have a significant glycocalyx extending from their apical surfaces throughout implantation, but the glycocalyx of the endometrial LE is always thicker than that of the trophoblast. The apical surface of the endometrial LE begins to physically protrude into the apical surface of the trophoblast cells on days 13 and 14, serving to immobilize the blastocyst that was previously free within the limited space of the uterine lumen. By days 15 and 16 of pregnancy, microvilli form between the closely opposed apical plasma membranes of the trophoblast and endometrial LE cells. Between days 15 and 20, the apical domes on endometrial LE develop cytoplasmic protrusions that extend between trophoblast cells to the luminal space, and interdigitation of microvilli between trophoblast and endometrial LE increases significantly through day 26 of pregnancy [131].

It is accepted that in all eutherian mammals, a temporary loss of MUC1, a prominent component of the apical surface glycocalyx of the endometrial LE with an extended carbohydrate configuration that physically inhibits attachment of the conceptus trophoblast to the endometrial LE, is required before there can be firm attachment of the conceptus trophoblast to the endometrial LE [132]. Therefore, attachment of conceptus trophoblast to the endometrial LE is predicated on down-regulation in the expression of MUC1 on endometrial LE, an event that is associated with a progesterone down-regulating progesterone receptors (PGR) in the endometrial LE [127,131,132,133,134]. The association between down-regulation of PGR and MUC1 in endometrial LE is strongly supported by the fact that MUC1 is lost at the apical surface of the endometrial LE of cyclic pigs given intramuscular injections of progesterone to down-regulate PGR [134].

Interactions between carbohydrates and lectins on the apical surfaces of trophoblast and endometrial LE cells during conceptus attachment for implantation have not been systematically investigated in pigs. However, loss of MUC1 from endometrial LE is believed to expose low affinity carbohydrate ligand binding molecules including selectins, galectins, heparan sulfate proteoglycan, heparin binding epidermal growth factor (EGF)-like growth factors, cadherins, and CD44 that are proposed to contribute to the initial attachment of conceptus trophoblast to the endometrial LE [117,135,136,137]. It is hypothesized that these carbohydrate ligands and their lectin receptors, expressed at the apical surfaces of the trophoblast and endometrial LE of pigs, mediate attach-and-release events between the respective cell types, similar to the “Rolling and Tethering” of leukocytes to the endothelium for extravasation into connective tissues [138], and similar to events proposed for the attachment blastocysts to the uterine wall of humans [139]. Indeed, goats and sheep express H-type-1 antigens and glycosylation dependent cell adhesion molecule 1 (GLYCAM1), respectively, at the interface between conceptus trophoblast and endometrial LE during blastocyst adhesion for implantation [137,140,141].

These proposed low affinity contacts between the apical surfaces of conceptus trophoblast and endometrial LE are stabilized by adhesion between a repertoire of integrins and their ECM ligands [115,120,125]. Integrin–ligand binding promotes cell–cell attachment, and integrins are major components of many adhesion cascades [120,142,143]. Integrins are transmembrane glycoprotein receptors composed of non-covalently linked α and β subunits, and the known 18 α- and 8 β-subunits can dimerize to form 24 heterodimer combinations that bind to numerous ECM proteins [143,144,145]. In pigs, α1, α3, α4, α5, αv, β1, β3, and β5 integrin subunits are expressed at the apical surface of both conceptus trophoblast and endometrial LE, and progesterone up-regulates the α4-, α5-, and β1-subunits on endometrial LE during the peri-implantation period [134]. The integrin subunits detected at the apical surface of the endometrial LE and conceptus trophoblast of pigs potentially assemble into the αvβ1, αvβ3, αvβ5, α4β1, and α5β1 integrin receptors, and have the opportunity to interact with ECM proteins expressed at the uterine–placental interface of pigs including (1) fibronectin (FN1) [134] which binds to αvβ3, α4β1, and α5β1, (2) vitronectin (VTN) [134] which binds to αvβ3, (3) the latency associated peptide (LAP) of transforming growth factor beta (TGFβ) [146] which binds to αvβ1 and αvβ3, (4) the inter-α-trypsin inhibitor heavy chain-like protein (IαIH4) [147] which binds to αvβ3, and/or (5) secreted phosphoprotein 1 (SPP1, also known as osteopontin or OPN) [148] which is up-regulated by E2 [149] and binds to αvβ1, αvβ3, αvβ5, α4β1 and α5β1. Affinity chromatography followed by immunoprecipitation has demonstrated direct binding of specific integrin receptors to ECM ligands on cultured endometrial epithelial (pUE) and conceptus trophoblast (pTr2) cells of pigs [150,151]. The integrin subunits αv, β1, β3, β5, β6, and β8 were shown to bind LAP [151], whereas the αvβ6 integrin receptor on cultured porcine trophoblast (pTr2) cells and αvβ3 integrin receptor on cultured porcine uterine epithelial (pUE) cells bound to OPN [150]. Integrin binding to OPN promoted dose-dependent attachment of pTr2 and pUE cells, and stimulated haptotactic pTr2 cell migration directionally along a physical gradient of non-soluble OPN [150]. Knockdown of the αv-subunit in pTr2 cells by siRNA reduced pTr2 attachment to OPN and FN1 [152], and the αv-subunit co-localizes with TLN1 in integrin adhesion complexes (IACs) generated at the apical domain of pTr2 cells around OPN-coated microspheres briefly cultured at the top of both cultured pig and sheep trophoblast cells [150,153].

## 6. Conceptus Implantation in Sheep and Cows

Figure 5 shows a site of conceptus implantation in the sheep from day 20 of pregnancy. What is understood about the “Adhesion Cascade for Implantation” in sheep has been reviewed in detail [6,12,13,120,137]. The electron micrographic studies of Guillomot (1981 and 1982) provide an excellent overview of the physical interactions between conceptus and endometrium during this period [19,20]. By day 14, the apical surface of elongating conceptus trophoblast cells are closely opposed to the apical surfaces of endometrial LE with the LE cells protruding into the trophoblast. At this time the conceptus is immobilized within the uterine lumen but can still be recovered intact from the uterus by lavage with only superficial damage. Between days 13 and 15, there is a reduction in the microvilli on trophoblast, but this is not observed for endometrial LE. By day 16, apposition begins near the ICM, and spreads towards the ends of the elongated conceptus with the development of cytoplasmic projections of trophoblast cells and endometrial LE microvilli that firmly adhere the conceptus to the uterus. Interdigitation of apical projections between conceptus trophoblast and endometrial LE occurs in both caruncular and intercaruncular regions of the uterus and is completed by Day 22 of pregnancy [19,20]. Between days 14 and 16, 15 to 20% of the trophoblast cells undergo consecutive nuclear divisions without cytokinesis to from binucleate trophoblast giant cells (TGCs) which express pregnancy-associated glycoproteins (PAGs) and fuse with endometrial LE to form syncytia. The extent of, and mechanistic details of the development of these syncytia continue to be investigated [57,59,154,155,156].

The current consensus for the cascade of events underlying attachment of the conceptus to the endometrium in sheep begins with down-regulation of MUC1 at the apical surface of endometrial LE cells across both caruncular and intercaruncular regions of the uterus. Unlike pigs, progesterone does not appear to down-regulate MUC1 expression on endometrial LE [134,153], but temporary loss of endometrial LE cells may in part be responsible for a decreased presence of MUC1 at the uterine–placental interface in ruminants (Figure 4C) [12,13,57,59,156]. MUC1 is a glycocalyx molecule that extends a substantial distance outward from the apical surface of endometrial LE cells. Its removal allows interactions between lectin and integrin receptors, present on the apical surfaces of the closely opposed conceptus trophoblast and endometrial LE, and their bridging ligands. Molecules that have been shown to potentially mediate attachment of conceptus trophoblast to endometrial LE in sheep include glycosylation dependent cell adhesion molecule 1 (GLYCAM-1), galectin 15 (LGALS15), OPN, and integrin receptors [120,125,137,141,153,157,158,159,160,161]. Initial attachment is likely mediated by GLYCAM1 and LGALS15, and firm attachment is likely mediated by OPN binding to integrin receptors [6,12,120,125,137,141,157,158,159,160,161]. LGALS15 expression is induced by progesterone and is further increased by IFNT [160], and OPN is induced by progesterone in the endometrial GE and secreted into the uterine lumen [157,158,159,162]. The integrin subunits αv, α4, α5, β1, β3, and β5 are constitutively present on the apical surfaces of conceptus trophoblast and endometrial LE cells and potentially form the integrin receptors αvβ3, αvβ1, αvβ5, α4β1, and α5β1 during the peri-implantation period of pregnancy [120,125,153,158].

There is evidence for integrins influencing early pregnancy in sheep. (1) Morpholino antisense oligonucleotides were used to block the translation of mRNA for the β3 integrin subunit in conceptus trophoblast and although the conceptuses elongated and attached to the endometrium the embryos were smaller than normal on day 25 and the allantois of the developing placenta had decreased levels of OPN and nitric oxide synthase 3 (NOS3), suggesting effects on development of the allantoic vasculature that transports nutrients to support growth and development of the embryo [163]. (2) The β5 integrin subunit extends from the apical surface of the endometrial LE into the sGE where it can interact with trophoblast papillae that likely serve as tethers against which forces necessary to generate elongation are applied, and serve to actively uptake histotroph [158]. (3) The integrin subunits αv, α4, β1, β3, and β5 are present at the apical surface of conceptus trophoblast and endometrial LE from days 11 through 16 implying roles for αvβ3, αvβ5, and α4β1 integrin receptors in attaching conceptus trophoblast to endometrial LE for implantation [153]. Affinity chromatography and immunoprecipitation experiments determined that the αvβ3 integrin receptor on cultured sheep trophoblast cells (oTr1) binds OPN [164]. It was further established that arginine-glycine-aspartate (RGD)-mediated interaction between integrins and OPN stimulate oTr1 cell adhesion, the αv integrin subunit incorporates into IACs around OPN-coated microbeads at the apical surface of oTr1 cells [164], integrins interact via the RGD sequence of OPN to activate mitogen-activated protein kinase (MAPK) p38 and p70 ribosomal protein S6 kinase beta-1 (P70S6K) [164], and IACs containing the β3 integrin subunit increase at the base of cultured oTr1 cells in response to treatment with the combination of arginine and OPN [165].

Figure 5 shows sites of conceptus implantation in cows from day 20 and day 31 of pregnancy. Little is known mechanistically about the “Adhesion Cascade for Implantation” in cows. The electron and light micrographic studies of King et al., (1980 and 1981) provide an excellent overview of the physical interactions between the conceptus and endometrium during this period [166,167]. By day 18 of gestation the height of the endometrial LE decreases as an initial response to the conceptus. Attachment of the conceptus trophoblast to the endometrial LE is first observed on day 20 with attachment occurring simultaneously in caruncular and intercaruncular regions of the uterus near the embryo and then spreading towards each end of the conceptus. Microvilli on the endometrial LE indent the apical membranes of the conceptus trophoblast cells. This develops into interdigitation between the microvilli of both conceptus trophoblast and endometrial LE by day 24, and intimate attachment by day 29 of pregnancy. Interestingly, large multinucleate cells were reported in the endometrial LE, and binucleate TGCs were present at these early stages of implantation. Placentomes are first recognizable on day 30 [166,167]. Alterations of the endometrial LE layer were subsequently reported to be limited to the formation of trinucleate syncytial cells formed through the fusion of TGCs with endometrial LE cells [168], but recent immunofluorescent staining has shown that syncytial cells with more than three nuclei are occasionally formed and expanses of the endometrial LE layer are temporally lost resulting in direct contact between trophoblast and the endometrial stroma which may explain why trophoblast-produced PAGs are reliable serum predictors of pregnancy in cows (Figure 4C) [12,60,169].

## 7. An Overview of Early Placental Development in Pigs, Sheep, and Cows

Firm adhesion of the conceptus to the endometrial LE provides a stable platform which is then subjected to subsequent morphological changes in the uterine–placental interface designed to optimize transport of nutrients from the mother to the fetus in these species with epitheliochorial and synepitheliochorial placentation. The uterine–placental interface of pigs progressively develops more complex folds that increase the surface area of contact between endometrium (consisting of the LE, stroma, and GE) and the placenta (consisting of the chorionic epithelium and allantois) for exchange of nutrients, gases and waste products [170,171]. The interface between the uterus and placenta of pigs begins to fold between day 20 and day 25 of pregnancy. These early folds increase in length through day 35, and again increase in length between days 50 and 60 of gestation (Figure 6A) [58]. These folds are likely the result of mechanotransduction and mechanosensation at the uterine–placental interface [58]. It is proposed that dilation of subepithelial uterine blood vessels delivers increased blood flow that pushes upward on the interface between the endometrium and the placental chorioallantois. These protrusive forces trigger IAC assembly and actin polymerization between the endometrial LE and chorionic epithelium at the tops of the uterine–placental folds. At the same time the endometrial stromal fibroblasts differentiate into contractile myofibroblasts that pull the connective tissue downward and inward to sculpt folds at the uterine–placental interface [58]. Placental and uterine capillaries lie immediately beneath these epithelia, minimizing the distance between maternal and fetal blood vessels [172]. Therefore, the increase in folding at the uterine (endometrial)-placental (chorioallantoic) interface increases the surface area of contact between maternal and placental micro-vasculatures to optimize the transport of nutrients from maternal to placental blood vessels for eventual utilization by the fetus. A detailed histological description of the developing uterine–placental folds is provided by Fries et al., (1980 and 1981), and again is summarized in Johnson et al., (2021), and will not be described in detail here [9,173,174]. The changing microanatomy of the uterine–placental interface profoundly influences gene expression in the different cell types found at this interface (Figure 6A). There are at minimum four different cell types that line the uterine–placental folds of pigs. These include tall columnar chorionic epithelial cells at the tops of the uterine–placental folds, cuboidal chorionic epithelial cells at the bottoms of the folds, nearly squamous endometrial LE cells at the tops of the folds, and low cuboidal endometrial LE cells at the bottoms of the folds. Each cell type exhibits a unique overall pattern of gene expression from the others, and some examples of differential gene expression are listed in the legend for Figure 6A.

In addition to having chorionic epithelium closely opposed to the endometrial LE, the placentae of pigs have specialized chorionic epithelial cells that line areolae at the openings of the mouths of endometrial GE (Figure 6A) [8,9,124,175]. The interplacentomal areas of the uterine–placental interface of sheep and cows also contain areolae that take up histotroph secreted by the endometrial GE for transport and release into the placental vasculature that rings the areola [8,10,12,13,175,176,177]. The chorionic epithelium of the areolae are tall columnar cells that have numerous vacuoles containing the secretions of endometrial GE (histotroph or uterine milk). Indeed, the open space between the chorion and endometrial LE is filled with histotroph [175]. The blood supply to the folds of the wall of the areolae form a ring towards the periphery and the areolar capillaries converge into one or two stem veins indicating facilitated external inflow of blood into the areola and outflow in a manner different from that of the inter-areolar regions of the placenta [178,179]. This microanatomy allows areolae to transport endometrial GE secretions such as macromolecules, particularly proteins, by fluid-phase pinocytosis across the placenta and into the fetal-placental circulation. Areolae begin to form as early as days 15 to 17 of gestation in pigs. The chorioallantois immediately around the openings of endometrial GE reaches over the mouth of the gland(s), develops a cavity that separates the endometrial LE from the chorioallantois, and a seal is formed between the chorionic epithelial cells and the opposed endometrial LE cells that line the placental border of this cavity to prevent dissipation of histotroph into inter-areolar regions of the placenta [174]. The balloon shape of the areola implies that there is interior pressure against the chorioallantoic surface of the areola delivered by the continuous accumulation of histotroph from the much larger endometrial GE [179]. There are about 2500 areolae distributed over the entire chorioallantois of the mature pig placenta with approximately 2.5 areolae per square centimeter of chorioallantois by the end of gestation [180].

Sheep and cows demonstrate synepitheliochorial placentation in which limited fusion of trophoblast with endometrial LE occurs [8,11,12,13]. Briefly, mononucleate trophoblast cells and binucleate trophoblast giant cells (TGCs), are present in the trophoblast of ruminant placentae. The TGCs begin to differentiate from the mononucleate trophoblast cells between days 14 and 16 and days 17 and 19 of gestation in sheep and cow conceptuses, respectively, and comprise 15–20% of the trophoblast during the apposition and adhesion phases of implantation. TGCs migrate and fuse with endometrial LE cells to form syncytial plaques in sheep, or syncytial cells in cows. In sheep, the syncytial plaques are a consistent feature in the placentomes throughout pregnancy [155]. It is noteworthy that the placentae of ruminants were once defined as syndesmochorial, because regions of direct contact between trophoblast and endometrial stroma were observed [181,182]. However, syndesmochorial placentation in sheep and cows has been rejected due to fact that the microanatomy of mature placentation in both species appears epitheliochorial in interplacentomal regions and synepitheliochorial in placentomes. Current histological studies suggest that the early stages of placental development in both sheep and cows are highly complex and may include limited regions of syndesmochorial placentation during, as yet, undetermined intervals of pregnancy (Figure 4C, Figure 5 and Figure 6B) [12,13,57,59,60,156]. Binucleate TGCs produce PAGs, and this expression is consistent enough to make concentrations of PAGs in the circulation of sheep and cows reliable markers of pregnancy [169,183,184,185,186,187,188]. Recent immunofluorescence microscopy examination of PAG expression in paraformaldehyde-fixed, paraffin-embedded, thin cross-sections of the intact uterine–placental interface of sheep and cows have identified (1) PAG positive cells not predicted to be present at the uterine–placental interface of sheep and cows, (2) limited regions of intense endometrial LE disruption at the uterine–placental interface of sheep and cows, and (3) E-cadherin positive cells in the stroma directly adjacent to these regions of endometrial LE disruption in sheep [57,59,60]. Figure 6B details some of the possible interpretations of what these data may mean physiologically, but much is clearly left to be learned about early placentation in sheep and cows.

## 8. Conclusions: A Listing of “Omic” Studies in Pigs, Sheep, and Cows That Can Be Used to Link the Physiology of Early Pregnancy in These Species Together

The livestock species, including pigs, sheep, and cows, have served as valuable comparative biomedical models for understanding physiology, endocrinology, cell biology, and immunology of pregnancy across mammalian species. Clearly the work reviewed here is beneficial to understanding how to mitigate suboptimal pregnancies and pregnancy loss in pigs and cows (the sheep as an animal model has provided valuable insights into pregnancy across the ruminant species), but also translates to human medicine. A major goal for research in human pregnancy is to eventually develop in vitro methodologies, i.e., “artificial wombs”, of sorts, to bridge the gap between the 14 days that conceptuses can be cultured and the 20 day lower limit of fetal viability. To conclude we provide a list of some publications of “omic” studies in pigs [189,190,191,192,193,194], sheep [194,195,196], and cows [197,198,199,200,201,202,203,204,205].

## Data Availability

The original contributions presented in this study are included in the article. Further inquiries can be directed to the corresponding author.

## References

[1] Wildeman D.E., Chen C., Erez O., Grossman L.I., Goodman M., Romero R. (2006). Evolution of the mammalian placenta revealed by phylogenetic analysis. Proc. Natl. Acad. Sci. USA.

[2] Forde N., Lonergan P. (2012). Transcriptomic Analysis of the Bovine Endometrium: What is Required to Establish Uterine Receptivity to Implantation in Cattle?. J. Reprod. Develop..

[3] Bazer F.W., Johnson G.A. (2014). Pig blastocyst-uterine interactions. Differentiation.

[4] Geisert R.D., Johnson G.A., Burghardt R.C. (2015). Implantation and establishment of pregnancy in the pig. Adv. Anat. Embryo. Cell Biol..

[5] Waclawik A., Kaczemarek M.M., Blitek A., Kaczynski P., Ziecik A.J. (2017). Embryo-maternal dialogue during pregnancy establishment and implantation in the pig. Mol. Reprod. Dev..

[6] Johnson G.A., Bazer F.W., Burghardt R.C., Wu G., Seo H., Kramer A.C., McLendon B.A. (2018). Cellular events during ovine implantation and impact for gestation. Anim. Reprod..

[7] Bazer F.W., Burghardt R.C., Johnson G.A., Spencer T.E., Wu G. (2018). Mechanisms for the establishment and maintenance of pregnancy: Synergies from scientific collaborations. Biol. Reprod..

[8] Johnson G.A., Skinner M.K. (2018). Domestic animal placentation. Encyclopedia of Reproduction.

[9] Johnson G.A., Bazer F.W., Seo H. (2021). The Early Stages of Implantation and Placentation in the Pig. Adv. Anat. Embryol. Cell Biol..

[10] Green J.A., Geisert R., Johnson G.A., Spencer T.E. (2021). Implantation and Placentation in Ruminants. Adv. Anat. Embrol. Cell Biol..

[11] Stenhouse C., Wu G., Seo H., Johnson G.A., Bazer F.W. (2022). Insights into the Regulation of Implantation and Placentation in Humans, Rodents, Sheep, and Pigs. Adv. Exp. Med. Biol..

[12] Johnson G.A., Bazer F.W., Seo H., Burghardt R.C., Wu G., Pohler K.G., Cain J.W. (2023). Understanding placentation in ruminants: A review focusing on cows and sheep. Reprod. Fertil. Develop..

[13] Davenport K.M., Ortega M.S., Johnson G.A., Seo H., Spencer T.E. (2023). Review: Implantation and placentation in ruminants. Animal.

[14] Kaczynski P., Goryszewska-Szczurek E., Baryla M., Waclawik A. (2023). Novel insight into conceptus-maternal signaling during pregnancy establishment in pigs. Mol. Reprod. Dev..

[15] Heuser C.H., Streeter G.L. (1929). Early stages in the development of pig embryos from the period of initial cleavage to the time of the appearance f limb buds. Contrib. Embryol. Carnegie Inst..

[16] Perry J.S., Rowlands I.W. (1962). Early pregnancy in the pig. J. Reprod. Fertil..

[17] Anderson L.L. (1978). Growth, protein content, and distribution of early pig embryos. Anat. Rec..

[18] Betteridge K.J., Eaglesome M.D., Randall G.C., Mitchell D. (1980). Collection, description and transfer of embryos from cattle 10–16 days after oestrus. J. Reprod. Fert..

[19] Guillomot M., Fléchon J.E., Wintenberger-Torres S. (1981). Conceptus attachment in the ewe: An ultrastructural study. Placenta.

[20] Guillomot M., Guay P. (1982). Ultrastructural features of the cell surfaces of uterine and trophoblastic epithelia during embryo attachment in the cow. Anatom. Rec..

[21] Geisert R.D., Brookbank J.W., Roberts R.M., Bazer F.W. (1982). Establishment of pregnancy in the pig. II. Cellular remodeling of the porcine blastocyst during elongation on day 12 of pregnancy. Biol. Reprod..

[22] Betteridge K.J. (1988). The anatomy and physiology of the pre-attachment bovine embryos. Theriogenology.

[23] Stroband H.W.J., Taverne N., Bogard M.V.D. (1989). The pig blastocyst: Its ultra-structure and the uptake of protein molecules. Cell Tissue Res..

[24] Wales R.G., Cuneo C.L. (1989). Morphology and Chemical Analysis of the Sheep Conceptus from the 13th to the 19th Day of Pregnancy. Reprod. Fertil. Develop..

[25] Mattson B.A., Overstrom E.W., Albertini D.F. (1990). Transitions in trophectoderm cellular shape and cytoskeletal organization in the elongating pig blastocyst. Biol. Reprod..

[26] Bazer F.W., Johnson G.A., Spencer T.E. (2005). Growth and development: Pre-implantation embryo. Encyclopedia of Animal Science.

[27] Spencer T.E., Johnson G.A., Bazer F.W., Burghardt R.C. (2007). Fetal-maternal interactions during the establishment of pregnancy in ruminants. Soc. Reprod. Fertil..

[28] Brooks K., Burns G., Spencer T.E. (2014). Conceptus elongation in ruminants: Roles of progesterone, prostaglandin, interferon tau and cortisol. J. Anim. Sci. Biotech..

[29] Bazer F.W., Seo H., Johnson G.A., Wu G. (2021). One-carbon metabolism and development of the conceptus during pregnancy: Lessons from studies with sheep and pigs. Amino Acids in Nutrition and Health.

[30] Seo H., Johnson G.A., Bazer F.W., Wu G., McLendon B.A., Kramer A.C. (2021). Cell-specific expression of enzymes required for serine biosynthesis and glutaminolysis in farm animals. Adv. Exp. Med. Biol..

[31] Seo H., Kramer A.C., McLendon B.A., Cain J.W., Burghardt R.C., Wu G., Bazer F.W., Johnson G.A. (2022). Elongating porcine coneptuses utilize glutaminolysis as an anaplerotic pathway to maintain the TCA cycle. Biol. Reprod..

[32] Moses R.M., Kramer A.C., Seo H., Wu G., Johnson G., Bazer F.W. (2022). A Role for Fructose Metabolism in Development of Sheep and Pig Conceptuses. Adv. Exp. Med. Biol..

[33] Johnson G.A., Seo H., Bazer F.W., Wu G., Kramer A.C., McLendon B.A., Cain J.W. (2023). Metabolic Pathways Utilized by the Porcine Conceptus, Uterus and Placenta. Mol. Reprod. Develop..

[34] Zavy M.T., Clark W.R., Sharp D.C., Roberts R.M., Bazer F.W. (1982). Comparison of glucose, fructose, ascorbic acid and glucosephosphate isomerase enzymatic activity in uterine flushings from nonpregnant and pregnant tils and pony mares. Biol. Reprod..

[35] Gao H., Wu G., Spencer T.E., Johnson G.A., Li X., Bazer F.W. (2009). Select nutrients in the ovine uterine lumen: I. amino acids, glucose and ions in uterine luminal fluid from cyclic and pregnant ewes. Biol. Reprod..

[36] Kramer A.C., Steinhauser C.B., Gao H., Seo H., McLendon B.A., Burghardt R.C., Wu G., Bazer F.W., Johnson G.A. (2020). Steroids Regulate Expression of SLC2A1 and SLC2A3 to Deliver Glucose into Trophectoderm for Metabolism via glycolysis. Endocrinology.

[37] Zhao F.Q., Keating A.F. (2007). Functional properties and genomics of glucose transporters. Curr. Genom..

[38] DeBosch B.J., Chen Z., Saben J.L., Finck B.N., Moley K.H. (2014). Glucose Transporter 8 (GLUT8) Mediates Fructose-induced de Novo Lipogenesis and Macrosteatosis. J. Biol. Chem..

[39] Steinhauser C.B., Landers M., Myatt L., Burghardt R.C., Vallet J.L., Bazer F.B., Johnson G.A. (2016). Fructose Synthesis and Transport at the Uterine-Placental Interface of Pigs: Cell-Specific Localization of SLC2A5, SLC2A8, and Components of the Polyol Pathway. Biol. Reprod..

[40] Gao H., Wu G., Spencer T.E., Johnson G.A., Bazer F.W. (2009). Select nutrients in the ovine uterine lumen: II. Glucose transporters in the uterus and peri-implantation conceptuses. Biol. Reprod..

[41] Wooding F.B., Fowden A.L., Bell A.W., Ehrhardt R.A., Limesand S.W., Hay W.W. (2005). Localisation of glucose transport in the ruminant placenta: Implications for sequential use of transporter isoforms. Placenta.

[42] Jeong W., Bazer F.W., Song G., Kim J. (2016). Expression of hypoxia-inducible factor-1 by trophectoderm cells in response to hypoxia and epidermal growth factor. Biochem. Biophys. Res. Commun..

[43] Burton G.J., Jauniaux E., Murray A.J. (2017). Oxygen and placental development; parallels and differences with tumour biology. Placenta.

[44] O’Neill L.A., Kishton R.J., Rathmell J. (2016). A guide to immunometabolism for immunologists. Nat. Rev. Immunol..

[45] McNamee E.N., Korns Johnson D., Homann D., Clambey E.T. (2013). Hypoxia and hypoxia-inducible factors as regulators of T cell development, differentiation, and function. Immunol. Rev..

[46] Bazer F.W., Wu G., Johnson G.A. (2024). Fructose metabolism is unregulated in cancers and placentae. Exp. Biol. Med..

[47] Moses R.M., Stenhouse C., Halloran K.M., Sah N., Hoskins E.C., Washburn S.E., Johnson G.A., Wu G., Bazer F.W. (2024). Metabolic pathways of glucose and fructose: I. Synthesis and metabolism of fructose by ovine conceptuses. Biol. Reprod..

[48] Moses R.M., Stenhouse C., Halloran K.M., Sah N., Newton M.G., Hoskins E.C., Washburn S.E., Johnson G.A., Wu G., Bazer F.W. (2024). Metabolic pathways of glucose and fructose: II. Spatiotemporal expression of genes involved in synthesis and transport of lactate in ovine conceptuses. Biol. Reprod..

[49] Kim J., Song G., Wu G., Bazer F.W. (2012). Functional roles of fructose. Proc. Natl. Acad. Sci. USA.

[50] Moses R.M., Halloran K.M., Stenhouse C., Sah N., Kramer A.C., McLendon B.A., Seo H., Johnson G.A., Wu G., Bazer F.W. (2022). Ovine conceptus tissue metabolizes fructose for metabolic support during the peri-implantation period of pregnancy. Biol. Reprod..

[51] Saxton R.A., Sabatini D.M. (2017). mTOR Signaling in Growth, Metabolism, and Disease. Cell.

[52] Halloran K.M., Stenhouse C., Moses R.M., Kramer A.C., Sah N., Seo H., Lamarre S.G., Johnson G.A., Wu G., Bazer F.W. (2023). The ovine conceptus utilizes extracellular serine, glucose and fructose to generate formate via the one carbon metabolism pathway. Amino Acids.

[53] Sah N., Stenhouse C., Halloran K.M., Moses R.M., Seo H., Burghardt R.C., Johnson G.A., Wu G., Bazer F.W. (2022). Inhibition of SHMT2 mRNA translation increases embryonic mortality in sheep. Biol. Reprod..

[54] Mullen A.R., Wheaton W.W., Jin E.S., Chen P.H., Sullivan L.B., Cheng T., Yang Y., Linehan W.M., Chandel N.S., DeBerardinis R.J. (2011). Reductive carboxylation supports growth in tumor cells with defective mitochondria. Nature.

[55] Yang L., Vennetim S., Nagrath D. (2017). Glutaminolysis: A hallmark of cancer metabolism. Annu. Rev. Biomed. Eng..

[56] Kim J., Song G., Wu G., Gao H., Johnson G.A., Bazer F.W. (2013). Arginine, leucine, and glutamine stimulate proliferation of porcine trophectoderm cells through the MTOR-RPS6K-RPS6-EIF4EBP1 signal transduction Pathway. Biol. Reprod..

[57] Seo H., Bazer F.W., Burghardt R.C., Johnson G.A. (2019). Immunohistochemical examination of trophoblast syncytialization during early placentation in sheep. Int. J. Mol. Sci..

[58] Seo H., Li X., Wu G., Bazer F.W., Burghardt R.C., Bayless K.J., Johnson G.A. (2020). Mechanotransduction drives morphogenesis to develop folding at the uterine-placental interface of pigs. Placenta.

[59] Seo H., Bazer F.W., Johnson G.A. (2024). Early Syncytialization of the Ovine Placenta Revisited. Results Probl. Cell Differ..

[60] Seo H., Melo G.D., Oliveira R.V., Franco-Johannsen F.A., Bazer F.W., Pohler K.G., Johnson G.A. (2024). Immunohistochemical examination of the utero-placental interface of cows on days 21, 31, 40, and 67 of gestation. Reproduction.

[61] Smith G.D., Takayama S., Swain J.E. (2012). Rethinking in Vitro Embryo Culture: New Developments in Culture Platforms and Potential to Improve Assisted Reproductive Technologies. Biol. Reprod..

[62] Heo Y.S., Cabrera L.M., Bormann C.L., Shah C.T., Takayama S., Smith G.D. (2010). Dynamic Microfunnel Culture Enhances Mouse Embryo Development and Pregnancy Rates. Hum. Reprod..

[63] Pranomphon T., López-Valiñas Á., Almiñana C., Mahé C., Brair V.L., Parnpai R., Mermillod P., Bauersachs S., Saint-Dizier M. (2024). Oviduct Epithelial Spheroids during in Vitro Culture of Bovine Embryos Mitigate Oxidative Stress, Improve Blastocyst Quality and Change the Embryonic Transcriptome. Biol. Res..

[64] Lai D., Ding J., Smith G.W., Smith G.D., Takayama S. (2015). Slow and Steady Cell Shrinkage Reduces Osmotic Stress in Bovine and Murine Oocyte and Zygote Vitrification. Hum. Reprod..

[65] Massip A., Mulnard J. (1980). Time-Lapse Cinematographic Analysis of Hatching of Normal and Frozen-Thawed Cow Blastocysts. J. Reprod. Fertil..

[66] Miles J.R., Walsh S.C., Rempel L.A., Pannier A.K. (2023). Mechanisms Regulating the Initiation of Porcine Conceptus Elongation. Mol. Reprod. Dev..

[67] Tinning H., Edge J.C., DeBem T.H.C., Deligianni F., Giovanardi G., Pensabene V., Meirelles F.V., Forde N. (2023). Review: Endometrial Function in Pregnancy Establishment in Cattle. Animal.

[68] Ferraz M.d.A.M.M., Ferronato G.d.A. (2023). Opportunities Involving Microfluidics and 3D Culture Systems to the in Vitro Embryo Production. Anim. Reprod..

[69] Simintiras C.A., Sánchez J.M., McDonald M., Lonergan P. (2019). The Biochemistry Surrounding Bovine Conceptus Elongation. Biol. Reprod..

[70] Gray C.A., Taylor K.M., Ramsey W.S., Hill J.R., Bazer F.W., Bartol F.F., Spencer T.E. (2001). Endometrial Glands Are Required for Preimplantation Conceptus Elongation and Survival. Biol. Reprod..

[71] Sargus-Patino C.N., Wright E.C., Plautz S.A., Miles J.R., Vallet J.L., Pannier A.K. (2014). In Vitro Development of Preimplantation Porcine Embryos Using Alginate Hydrogels as a Three-Dimensional Extracellular Matrix. Reprod. Fertil. Dev..

[72] Laughlin T.D., Miles J.R., Wright-Johnson E.C., Rempel L.A., Lents C.A., Pannier A.K. (2017). Development of Pre-Implantation Porcine Blastocysts Cultured within Alginate Hydrogel Systems Either Supplemented with Secreted Phosphoprotein 1 or Conjugated with Arg-Gly-Asp Peptide. Reprod. Fertil. Dev..

[73] Zhao S., Liu Z.X., Gao H., Wu Y., Fang Y., Wu S.S., Li M.J., Bai J.H., Liu Y., Evans A. (2015). A Three-Dimensional Culture System Using Alginate Hydrogel Prolongs Hatched Cattle Embryo Development in Vitro. Theriogenology.

[74] Brandão D.O., Maddox-Hyttel P., Løvendahl P., Rumpf R., Stringfellow D., Callesen H. (2004). Post Hatching Development: A Novel System for Extended in Vitro Culture of Bovine Embryos. Biol. Reprod..

[75] Ramos-Ibeas P., Lamas-Toranzo I., Martínez-Moro Á., de Frutos C., Quiroga A.C., Zurita E., Bermejo-Álvarez P. (2020). Embryonic Disc Formation Following Post-Hatching Bovine Embryo Development in Vitro. Reproduction.

[76] Vajta G., Alexopoulos N.I., Callesen H. (2004). Rapid Growth and Elongation of Bovine Blastocysts in Vitro in a Three-Dimensional Gel System. Theriogenology.

[77] Squires T.M. (2005). Microfluidics: Fluid Physics at the Nanoliter Scale. Rev. Mod. Phys..

[78] Wang H., Pilla F., Anderson S., Martínez-Escribano S., Herrer I., Moreno-moya J.M., Musti S., Bocca S., Oehninger S., Horcajadas J.A. (2012). A Novel Model of Human Implantation: 3D Endometrium-like Culture System to Study Attachment of Human Trophoblast (Jar) Cell Spheroids. Mol. Hum. Reprod..

[79] Haeger J.D., Hambruch N., Dilly M., Froehlich R., Pfarrer C. (2011). Formation of Bovine Placental Trophoblast Spheroids. Cells Tissues Organs.

[80] Aisenbrey E.A., Murphy W.L. (2020). Synthetic Alternatives to Matrigel. Nat. Rev. Mater..

[81] Ferraz M.A.M.M., Rho H.S., Hemerich D., Henning H.H.W., van Tol H.T.A., Hölker M., Besenfelder U., Mokry M., Vos P.L.A.M., Stout T.A.E. (2018). An Oviduct-on-a-Chip Provides an Enhanced in Vitro Environment for Zygote Genome Reprogramming. Nat. Commun..

[82] Ferraz M.A.M.M., Henning H.H.W., Costa P.F., Malda J., Melchels F.P., Wubbolts R., Stout T.A.E., Vos P.L.A.M., Gadella B.M. (2017). Improved Bovine Embryo Production in an Oviduct-on-a-Chip System: Prevention of Poly-Spermic Fertilization and Parthenogenic Activation. Lab Chip.

[83] Sugino Y., Sato T., Yamamoto Y., Kimura K. (2022). Evaluation of Bovine Uterine Gland Functions in 2D and 3D Culture System. J. Reprod. Dev..

[84] Nishino D., Kotake A., Yun C.S., Rahman A.N.M.I., El-Sharawy M., Yamanaka K.I., Khandoker M.A.M.Y., Yamauchi N. (2021). Gene Expression of Bovine Endometrial Epithelial Cells Cultured in Matrigel. Cell Tissue Res..

[85] Kitamura A., Hori T., Nashimoto Y., Nakato R., Hamada H., Kaji H., Kikutake C., Suyama M., Saito M., Yaegashi N. (2024). Modeling Embryo-Endometrial Interface Recapitulating Human Embryo Implantation. Sci. Adv..

[86] Sakurai T., Bai H., Bai R., Arai M., Iwazawa M., Zhang J., Konno T., Godkin J.D., Okuda K., Imakawa K. (2012). Coculture System That Mimics in Vivo Attachment Processes in Bovine Trophoblast Cells. Biol. Reprod..

[87] Yamakoshi S., Bai R., Chaen T., Ideta A., Aoyagi Y., Sakurai T., Konno T., Imakawa K. (2012). Expression of Mesenchymal-Related Genes by the Bovine Trophectoderm Following Conceptus Attachment to the Endometrial Epithelium. Reproduction.

[88] Cain J.W., Lefevre C., Ross A., Johnson G.A., Norris D.O., Lopez. K.H. (2024). Hormones and reproductive cycles in ungulates. Hormones and Reproduction of Vertebrates.

[89] Lefèvre F., Guillomot M., D’Andrea S., Battegay S., La Bonnardière C. (1988). Interferon-delta: The first member of a novel type I interferon family. Biochimie.

[90] La Bonnardière C., Martinat-Botté F., Terqui M., Lefèvre F., Zouari K., Martal J., Bazer F.W. (1991). Production of two species of interferon by Large White and Meishan pig conceptuses during the peri-attachment period. J. Reprod. Fert..

[91] Johnson G.A., Bazer F.W., Burghardt R.C., Spencer T.E., Wu G., Bayless K.J. (2009). Conceptus-uterus interactions in pigs: Endometrial gene expression in response to estrogens and interferons from conceptuses. Soc. Reprod. Fertil. Suppl..

[92] Harney J.P., Bazer F.W. (1989). Effect of porcine conceptus secretory proteins on interestrous interval and uterine secretion of prostaglandins. Biol. Reprod..

[93] Lefèvre F., Martinat-Botté F., Locatelli A., De Niu P., Terqui M., La Bonnardière C. (1988). Intrauterine infusion of high doses of pig trophoblast interferons has no antiluteolytic effect in cyclic gilts. Biol. Reprod..

[94] Geisert R.D., Johns D.N., Pfeiffer C.A., Sullivan R.M., Lucas C.G., Simintiras C.A., Redel B.K., Wells K.D., Spencer T.E., Prather R.S. (2023). Gene editing provides a tool to investigate genes involved in reproduction of pigs. Mol. Reprod. Dev..

[95] Geisert R.D., Bazer F.W., Lucas C.G., Pfeiffer C.A., Meyer A.E., Sullivan R., Johns D.N., Sponchiado M., Prather R.S. (2024). Maternal recognition of pregnancy in the pig: A servomechanism involving sex steroids, cytokines and prostaglandins. Anim. Reprod. Sci..

[96] Cain J.W., Seo H., Bumgardner K., Lefever C., Burghardt R.C., Bazer F.W., Johnson G.A. (2024). Pig conceptuses release extracelluar vesicles containing IFNG for paracrine communication with the endometrium. Biol. Reprod..

[97] Joyce M.M., Burghardt R.C., Geisert R.D., Burghardt J.R., Hooper R.N., Ross J.W., Ashworth M.D., Johnson G.A. (2007). Pig conceptuses secrete estrogen and interferons to differentially regulate uterine STAT1 in a temporal and cell-type specific manner. Endocrinology.

[98] Joyce M.M., Burghardt J.R., Burghardt R.C., Hooper R.N., Jaeger L.A., Spencer T.E., Bazer F.W., Johnson G.A. (2007). Pig conceptuses increase uterine interferon regulatory factor-1 (IRF-1), but restrict expression to stroma through estrogen-induced IRF-2 in luminal epithelium. Biol. Reprod..

[99] Joyce M.M., Burghardt J.R., Burghardt R.C., Hooper R.N., Bazer F.W., Johnson G.A. (2008). Uterine major histocompatibility class I molecules and beta 2 microglobulin are regulated by progesterone and conceptus interferons during pig pregnancy. J. Immunol..

[100] McLendon B.A., Seo H., Kramer A.C., Burghardt R.C., Bazer F.W., Johnson G.A. (2020). Pig conceptuses secrete interferon gamma to recruit T cells to the endometrium during the peri-implantation period. Biol. Reprod..

[101] Johns D.N., Lucas C.G., Pfeiffer C.A., Chen P.R., Meyer A.E., Perry S.C., Spate L.D., Cecil R.F., Fudge M.A., Samuel M.S. (2021). Conceptus interferon gamma is essential for establishment of pregnancy in the pig. Riol. Reprod..

[102] Hansen T.R., Austin K.J., Johnson G.A. (1997). Transient ubiquitin cross-reactive protein gene expression in bovine endometrium. Endocrinology.

[103] Johnson G.A., Austin K.J., Collins A.M., Murdoch W.J., Hansen T.R. (1999). Endometrial ISG17 mRNA and a related mRNA are induced by interferon-tau and localized to glandular epithelial and stromal cells from pregnant cows. Endocrine.

[104] Joyce M.M., White F.J., Burghardt R.C., Muñiz J.J., Spencer T.E., Bazer F.W., Johnson G.A. (2005). Interferon stimulated gene 15 (ISG15) conjugates to cytosolic proteins and is expressed at the uterine-placental interface throughout ovine pregnancy. Endocrinology.

[105] Johnson G.A., Bazer F.W., Burghardt R.C., Seo H., Wu G., Cain J.W., Pohler K.G. (2024). The history of interferon stimulated genes (ISGs) in pregnant cattle, sheep, and pigs. Reproduction.

[106] Ruiz-Gonzalez I., Xu J., Wang X., Burghardt R.C., Dunlap K.A., Bazer F.W. (2015). Exosomes, endogenous retroviruses and toll-like receptors: Pregnancy recognition in ewes. Reproduction.

[107] Burns G.W., Brooks K.E., Spencer T.E. (2016). Extracellular vesicles originate from the conceptus and uterus during early pregnancy in sheep. Biol. Reprod..

[108] Godkin J.D., Bazer F.W., Thatcher W.W., Roberts R.M. (1984). Proteins released by cultured day 15–16 conceptuses prolong luteal maintenance when introduced into the uterine lumen of cyclic ewes. J. Reprod. Fertil..

[109] Spencer T.E., Becker W.C., George P., Mirando M.A., Ogle T.F., Bazer F.W. (1995). Ovine interferon-τ regulates expression of endometrial receptors for estrogen and oxytocin by not progesterone. Biol. Reprod..

[110] Wathes D.C., Lamming G.E. (1995). The oxytocin receptor, luteolysis and the maintenance of pregnancy. J. Reprod. Fertil. Suppl..

[111] Fleming J.G.W., Spencer T.E., Safe S.S., Bazer F.W. (2006). Estrogen regulates transcription of the ovine oxytocin receptor gene through GC-rich SP1 promoter elements. Endocrinology.

[112] Bazer F.W., Johnson G.A., Burghardt R.C., Pond W.G., Bell A.W. (2005). Implantation. Encyclopedia of Animal Science.

[113] Denker H.W. (1993). Implantation: A cell biological paradox. J. Exp. Zool..

[114] Guillomot M. (1995). Cellular interactions during implantation in domestic ruminants. J. Reprod. Fertil..

[115] Burghardt R.C., Johnson G.A., Jaeger L.A., Ka H., Garlow J.E., Spencer T.E., Bazer F.W. (2022). Integrins and extracellular matrix proteins at the maternal/fetal interface in domestic animals. Cells Tissues Organs.

[116] Aplin J.D., Meseguer M., Simon C., Ortiz M.E., Croxatto H., Jones C.J. (2001). MUC1, glycans and the cell-surface barrier to embryo implantation. Biochem. Soc. Trans..

[117] Kimber S.J., Illingworth I.M., Glasser S.R. (1995). Expression of carbohydrate antigens in the rat uterus during early pregnancy and after ovariectomy and steroid replacement. J. Reprod. Fertil..

[118] Lessey B.A. (2002). Adhesion molecules and implantation. J. Reprod. Immunol..

[119] Aplin J.D., Kimber S.J. (2004). Trophoblast-uterine interactions at implantation. Reprod. Biol. Endocrinol..

[120] Johnson G.A., Burghardt R.C., Bazer F.W., Seo H., Cain J.W. (2023). Integrins and their potential roles in mammalian pregnancy. J. Anim. Sci. Biotech..

[121] Burton G.J. (2022). Placental types. Benirschke’s Pathology of the Human Placenta.

[122] Roberts R.M., Green J.A., Schulz L.C. (2016). The evolution of the placenta. Reproduction.

[123] Lefevre C.M., Cain J.W., Kramer A.C., Seo H., Lopez A.N., Sah N., Wu G., Bazer F.W., Johnson G.A. (2024). Evidence for metabolism of creatine by the conceptus, placenta, and uterus for production of ATP during conceptus development in pigs. Biol. Reprod..

[124] Song G., Bailey D.W., Dunlap K.A., Burghardt R.C., Spencer T.E., Bazer F.W., Johnson G.A. (2010). Cathepsin B, Cathepsin L and Cystatin C in the Porcine Uterus and Placenta: Potential Roles in Endometrial/Placental Remodeling and in Fluid-Phase Transport of Proteins Secreted by Uterine Epithelia Across Placental Areolae and Neonatal Gut. Biol. Reprod..

[125] Johnson G.A., Burghardt R.C., Bazer F.W. (2014). Osteopontin: A leading candidate adhesion molecule for implantation in pigs and sheep. J. Anim. Sci. Biotechnol..

[126] Bailey D.W., Dunlap K.L., Erikson D.W., Patel A., Bazer F.W., Burghardt R.C., Johnson G.A. (2010). Effects of Long-Term Progesterone Exposure on Porcine Uterine Gene Expression: Progesterone Alone Does Not Induce Secreted Phosphoprotein 1 (Osteopontin) in Glandular Epithelium. Reproduction.

[127] Steinhauser C.B., Bazer F.B., Burghardt R.C., Johnson G.A. (2017). Expression of Progesterone Receptor in the Porcine Uterus and Placenta throughout Gestation: Correlation with Expression of Uteroferrin and Osteopontin. Domestic. Anim. Endocrinol..

[128] McLendon B.A., Kramer A.C., Seo H., Burghardt R.C., Bazer F.W., Wu G., Johnson G.A. (2022). Temporal and spatial expression of aquaporins 1, 5, 8, and 9: Potential transport of water across the endometrium and chorioallantois of pigs. Placenta.

[129] Wu G., Li X., Seo H., McLendon B.A., Kramer A.C., Bazer F.W., Johnson G.A. (2022). Osteopontin (OPN)/Secreted Phosphoprotein 1 (SPP1) binds integrins to activate transport of ions across the porcine placenta. Front. Biosci..

[130] Bailey D.W., Dunlap K.A., Frank J.W., Erikson D.W., White B.G., Bazer F.W., Burghardt R.C., Johnson G.A. (2010). Effects of long-term progesterone on developmental and functional aspects of porcine uterine epithelia: Progesterone alone does not support glandular development of pregnancy. Reproduction.

[131] Dantzer V. (1985). Electron microscopy of the initial stages of placentation in the pig. Anat. Embryol..

[132] Brayman M., Thathiah A., Carson D.D. (2004). MUC1: A multifunctional cell surface component of reproductive tissue epithelia. Reprod. Biol. Endocrinol..

[133] Geisert R.D., Pratt T.N., Bazer F.W., Mayes J.S., Watson G.H. (1994). Immunocytochemical localization and changes in endometrial progestin receptor protein during the porcine oestrous cycle and early pregnancy. Reprod. Fertil. Dev..

[134] Bowen J.A., Bazer F.W., Burghardt R.C. (1996). Spatial and temporal analysis of integrin and Muc-1 expression in porcine uterine epithelium and trophectoderm in vivo. Biol. Reprod..

[135] Bowen J.A., Newton G.R., Weise D.W., Bazer F.W., Burghardt R.C. (1996). Characterization of a polarized porcine uterine epithelial model system. Biol. Reprod..

[136] Kimber S.J., Spanswick C. (2000). Blastocyst implantation: The adhesion cascade. Semin. Cell Dev. Biol..

[137] Spencer T.E., Johnson G.A., Bazer F.W., Burghardt R.C. (2004). Implantation mechanisms: Insights from the sheep. Reproduction.

[138] Kling D., Fingerle J., Harlan J.M. (1992). Inhibition of leukocyte extravasation with a monoclonal antibody to CD18 during formation of experimental intimal thickening in rabbit carotid arteries. Arterioscler. Thromb..

[139] Red-Horse K., Zhou Y., Genbacev O., Prakobphol A., Foulk R., McMaster M., Fisher S.J. (2004). Trophoblast differentiation during embryo implantation and formation of the maternal-fetal interface. J. Clin. Investig..

[140] Powell J.K., Glasser S.R., Woldesenbet S., Burghardt R.E., Newton G.R. (2000). Expression of carbohydrate antigens in the goat uterus during early pregnancy and on steroid-treated polarized uterine epithelial cells in vitro. Biol. Reprod..

[141] Spencer T.E., Bartol F.F., Bazer F.W., Johnson G.A., Joyce M.M. (1999). Identification and characterization of glycosylation dependent cell adhesion molecule 1 (GlyCAM-1) expression in the ovine uterus. Biol. Reprod..

[142] Giancotti F.G., Ruoslahti E. (1990). Integrin signaling. Science.

[143] Burghardt R.C., Burghardt J.R., Taylor II J.D., Reeder A.T., Nguyen B.T., Spencer T.E., Johnson G.A. (2009). Enhanced focal adhesion assembly reflects increased mechanosensation and mechanotransduction along the maternal/conceptus interface during pregnancy in sheep. Reproduction.

[144] Gallant N.D., Michael K.E., García A.J. (2005). Cell adhesion strengthening: Contributions of adhesive area, integrin binding, and focal adhesion assembly. Mol. Biol. Cell.

[145] Humphries J.D., Byron A., Humphries M.J. (2006). Integrin ligands at a glance. J. Cell Sci..

[146] Gupta A., Dekaney C.M., Bazer F.W., Madrigal M.M., Jaeger L.A. (1998). Beta transforming growth factors (TGFβs) at the porcine conceptus-maternal interface. Part II: Uterine TGFβ bioactivity and expression of immunoreactive TGFβs (TGFβ1, TGFβ2, and TGFβ3) and their receptors (Type I and Type II). Biol. Reprod..

[147] Geisert R.D., Yelich J.V., Pratt T., Pomp D. (1998). Expression of an inter-α-trypsin inhibitor heavy chain-like protein in the pig endometrium during the oestrous cycle and early pregnancy. J. Reprod. Fert..

[148] Garlow J.E., Ka H., Johnson G.A., Burghardt R.C., Jaeger L.A., Bazer F.W. (2002). Analysis of osteopontin at the maternal-placental interface in pigs. Biol. Reprod..

[149] White F.J., Ross J.W., Joyce M.M., Geisert R.D., Burghardt R.C., Johnson G.A. (2005). Steroid regulation of cell specific secreted phosphoprotein 1 (osteopontin) expression in the pregnant porcine uterus. Biol. Reprod..

[150] Erikson D.W., Burghardt R.C., Bayless K.J., Johnson G.A. (2009). Secreted phosphoprotein 1 (SPP1, osteopontin) binds to integrin alphavbeta6 on porcine trophectoderm cells and integrin alphavbeta3 on uterine luminal epithelial cells, and promotes trophectoderm cell adhesion and migration. Biol. Reprod..

[151] Massuto D.A., Kneese E.C., Johnson G.A., Hooper N.H., Burghardt R.C., Ing N.H., Jaeger L.A. (2009). Transforming growth factor beta (TGFB) signaling is activated during porcine implantation: Proposed role for latency associated peptide-integrins at the conceptus-maternal interface. Reproduction.

[152] Frank J.W., Seo H., Burghardt R.C., Bayless K.J., Johnson G.A. (2017). ITGAV (Alpha V Integrins) Bind SPP1 (Osteopontin) to Support Trophoblast Cell Adhesion. Reproduction.

[153] Johnson G.A., Bazer F.W., Jaeger L.A., Ka H., Garlow J.E., Pfarrer C., Spencer T.E., Burghardt R.C. (2001). Muc-1, integrin and osteopontin expression during the implantation cascade in sheep. Biol. Reprod..

[154] Wooding F.B., Flint A.P., Heap R.B., Morgan G., Buttle H.L., Young I.R. (1986). Control of binucleate cell migration in the placenta of sheep and goats. J. Reprod. Fertil..

[155] Wooding F.B.P. (2022). The ruminant placental trophoblast binucleate cell: An evolutionary breakthrough. Biol. Reprod..

[156] Seo H., Frank J.W., Burghardt R.C., Bazer F.W., Johnson G.A. (2020). Integrins and OPN localize to adhesion complexes during placentation in sheep. Reproduction.

[157] Johnson G.A., Spencer T.E., Burghardt R.C., Bazer F.W. (1999). Ovine Osteopontin: I. Cloning and expression of mRNA in the uterus during the peri-implantation period. Biol. Reprod..

[158] Johnson G.A., Burghardt R.C., Spencer T.E., Newton G.R., Ott T.L., Bazer F.W. (1999). Ovine Osteopontin: II. Osteopontin and α_v_β_3_ integrin expression in the uterus and conceptus during the peri-implantation period. Biol. Reprod..

[159] Johnson G.A., Burghardt R.C., Bazer F.W., Spencer T.E. (2003). Minireview: Osteopontin: Roles in implantation and placentation. Biol. Reprod..

[160] Gray C.A., Adelson D.L., Bazer F.W., Burghardt R.C., Meeusen E.N., Spencer T.E. (2004). Discovery and characterization of an epithelial-specific galectin in the endometrium that forms crystals in the trophectoderm. Proc. Natl. Acad. Sci. USA.

[161] Muñiz J.J., Joyce M.M., Taylor J.D., Burghardt J.R., Burghardt R.C., Johnson G.A. (2006). Glycosylation Dependent Cell Adhesion Molecule 1 (GlyCAM-1)-like protein and L-Selectin expression in sheep interplacentomal and placentomal endometrium. Reproduction.

[162] Johnson G.A., Spencer T.E., Burghardt R.C., Taylor K.M., Gray C.A., Bazer F.W. (2000). Progesterone modulation of osteopontin gene expression in the ovine uterus. Biol. Reprod..

[163] Frank J.W., Steinhauser C.B., Wang X., Bughardt R.C., Bazer F.W., Johnson G.A. (2021). Loss of ITGB3 in ovine conceptuses decreases conceptus expression of NOS3 and SPP1: Implications for the developing placental vasculature. Biol. Reprod..

[164] Kim J., Erikson D.W., Burghardt R.C., Spencer T.E., Wu G., Bayless K.J., Johnson G.A., Bazer F.W. (2010). Secreted phosphoprotein 1 binds integrins to initiate multiple cell signaling pathways, including FRAP1/mTOR, to support attachment and force-generated migration of trophectoderm cells. Matrix Biol..

[165] Wang X., Johnson G.A., Burghardt R.C., Wu G., Bazer F.W. (2016). Uterine Histotroph and Conceptus Development. II. Arginine and Secreted Phosphoprotein 1 Cooperatively Stimulate Migration and Adhesion of Ovine Trophectoderm Cells via Focal Adhesion-MTORC2 mediated Cytoskeleton Reorganization. Biol. Reprod..

[166] King G.J., Atkinson B.A., Robertson H.A. (1980). Development of the bovine placentome from days 20 to 29 of gestation. J. Reprod. Fertil..

[167] King G.J., Atkinson B.A., Robertson H.A. (1981). Development of the intercaruncular areas during early gestation and establishment of the bovine placenta. J. Reprod. Fertil..

[168] Wooding F.B. (1982). The role of the binucleate cell in ruminant placental structure. J. Reprod. Fertil..

[169] Reese S.T., Pereira M.H.C., Edwards J.L., Vasconcelos J.L.M., Pohler K.G. (2018). Pregnancy diagnosis in cattle using pregnancy associated glycoprotein concentration in circulation at day 24 of gestation. Theriogenology.

[170] Vallet J.L., Freking B.A. (2007). Differences in placental structure during gestation associated with large and small pig fetuses. J. Anim. Sci..

[171] Vallet J.L., Miles J.R., Freking B.A. (2009). Development of the pig placenta. Soc. Reprod. Fertil. Suppl..

[172] Dantzer V., Leiser R. (1994). Initial vascularisation in the pig placenta: I. Demonstration of nonglandular areas by histology and corrosion casts. Anat. Rec..

[173] Friess A.E., Sinowatz F., Skolek-Winnisch R., Traautner W. (1980). The placenta of the pig. I. Finestructural changes of the placental barrier during pregnancy. Anat. Embryol..

[174] Friess A.E., Sinowatz F., Skolek-Winnisch R., Traautner W. (1981). The placenta of the pig. II. Finestructural changes of the placental barrier during pregnancy. Anat. Embryol..

[175] Dempsey F.W., Wislocki B., Amoroso E.C. (1955). Electron microscopy of the pig’s placenta, with especial reference to the cell membranes of the endometrium and chorion. Am. J. Anat..

[176] Wooding F.B., Burton G.J. (2008). Chapter 6, Synepitheliochorial placentation: Ruminants (ewe and cow). Comparative Placentation: Structure, Function and Evolution.

[177] Assis Neto A.C., Pereira F.T.V., Santos T.C., Ambrosio C.E., Leiser R., Miglino M.A. (2010). Morpho-physical recording of bovine conceptus (*Bos indicus*) and placenta from days 20 to 70 of pregnancy. Reprod. Domest. Anim..

[178] Dantzer V., Leiser R. (1993). Microvascularization of regular and irregular areolae of the areola-gland submunit of the porcine placenta: Structural and functional aspects. Anat. Embryol..

[179] Leiser R., Dantzer V. (1994). Initial vascularization in the pig placenta: II. Demonstration of gland and areola-gland subunits by histology and corrosion casts. Anat. Rec..

[180] Knight J.W., Bazer F.W., Thatcher W.W., Franke D.E., Wallace H.D. (1977). Conceptus development in intact and unilaterally hysterectomized ovariectomized gilts: Interrelations among hormonal status, placental development, fetal fluids and fetal growth. J. Anim. Sci..

[181] Grosser O. (1909). Vergleichende Anatomie und Entwicklungsgeschichte der Eihaute und der Placenta mit Besonderer Berücksichtigung des Menschen.

[182] Grosser O. (1927). Fruhentwicklung, Eihautbidung und Placentation des Menschen und der Saugetiere.

[183] Sasser R.G., Ruder C.A., Ivani K.A., Butler J.E., Hamilton W.C. (1986). Detection of pregnancy by radioimmunoassay of a novel pregnancy-specific protein in serum of cows and a profile of serum concentrations during gestation. Biol. Reprod..

[184] Ruder C.A., Stellflug J.N., Dahmen J.J., Sasser R.G. (1988). Detection of pregnancy in sheep by radioimmunoassay of sera for pregnancy-specific protein B. Theriogenology.

[185] Karen A., Beckers J.F., Sulon J., de Sousa N.M., Szabados K., Reczigel J., Szenci O. (2003). Early pregnancy diagnosis in sheep by progesterone and pregnancy-associated glycoprotein tests. Theriogenology.

[186] Pohler K.G., Geary T.W., Johnson C.L., Atkins J.A., Jinks E.M., Busch D.C., Green J.A., MacNeil M.D., Smith M.F. (2013). Circulating bovine pregnancy associated glycoproteins are associated with late embryonic/fetal survival but not ovulatory follicle size in suckled beef cows. J. Anim. Sci..

[187] Wallace R.M., Pohler K.G., Smith M.F., Green J.A. (2015). Placental PAGs: Gene origins, expression patterns, and use as markers of pregnancy. Reproduction.

[188] Pohler K.G., Pereira M., Lopes F.R., Lawrence J.C., Keisler D.H., Smith M.F., Vasconcelos J., Green J.A. (2016). Circulating concentrations of bovine pregnancy-associated glycoproteins and late embryonic mortality in lactating dairy herds. J. Dairy Sci..

[189] Samborski A., Graf A., Krebs A., Kessler S., Bauersachs S. (2013). Deep sequencing of the porcine endometrial transcriptome on day 14 of pregnancy. Biol. Reprod..

[190] Samborski A., Graf A., Krebs A., Kessler S., Reichenbach M., Reichenbach H.D., Ulbrich S.E., Bauersachs S. (2013). Transcriptome changes in the porcine endometrium during the preattachement phase. Biol. Reprod..

[191] Kaczynski P., Bauersachs S., Baryla S., Goryszewska E., Muszak J., Grzegorzewski W.J., Waclawik A. (2020). Eatradiol-17β-induced changes in the porcine endometrial transcriptome in vivo. Int. J. Mol. Sci..

[192] Zang X., Gu T., Hu Q., Xu Z., Xie Y., Zhou C., Zheng E., Huang S., Xu Z., Fanming M. (2021). Global transcriptomic analyses reveal genes involved in conceptus development during the implantation stages in pigs. Front. Genet..

[193] Tian Q., He J.-P., Zhu C., Zhu Q.-Y., Li Y.-G., Liu J.-I. (2022). Revisiting the transcriptome landscape of pig embryo implantation site at single-cell resolution. Front. Cell Dev. Biol..

[194] Brooks K., Burns G.W., Moraes J.G.N., Spencer T.E. (2016). Analysis of the uterine epithelial and conceptus transcriptome and luminal fluid proteome during the peri-implantation period of pregnancy in sheep. Biol. Reprod..

[195] Matrsuno Y., Kusama K., Imakawa K. (2022). Characterization of lncRNA functioning in ovine conceptuses and endometria during the peri-implantation period. Biochem. Biophy. Res. Commun..

[196] Jia G.-X., Ma W.-J., Wu Z.-B., Li S., Zhang X.-Q., He Z., Wu S.-X., Tao H.-P., Fang Y., Song Y.-W. (2023). Single-cell transcriptomic characterization of sheep conceptus elongation and implantation. Cell Rep..

[197] Bauersachs S., Ulbrich S.E., Gross K., Schmidt S.E., Meyer H.H., Wenigerkind H., Vermehren M., Sinowatz F., Blum H., Wolf E. (2006). Embryo-induced transcriptome changes in bovine endometrium reveal species-specific and common molecular markers of uterine receptivity. Reproduction.

[198] Bauersachs S., Mitko K., Ulbrich S.E., Blum H., Wolf E. (2008). Transcriptome studies of bovine endometrium reveal molecular profiles characteristic for specific stages of estrous cycle and early pregnancy. Exp. Clin. Endocrinol. Diabetes.

[199] Forde N., Carter F., Spencer T.E., Bazer F.W., Sandra O., Mansouri-Attia N., Okumu L.A., McGettigan P.A., Mehta J.P., McBride R. (2011). Conceptus-induced changes in the endometrial transcriptome: How soon does the cow know she is pregnant?. Biol. Reprod..

[200] Biase F.H., Rabel C., Guillomot M., Hue I., Andropolis K., Olmstead C.A., Oliveira R., Wallace R., Le Bourhis D.L., Richard C. (2016). Massive dysregulation of genes involved in cell signaling an dplacental development in cloned cattle conceptus and maternal endometrium. Proc. Natl. Acad. Sci. USA.

[201] Moraes J.G.N., Behura S.K., Geary T.S., Hansen P.J., Neibergs H.L., Spencer T.E. (2018). Uterine influences on conceptus development in fertility-classified animals. Proc. Natl. Acad. Sci. USA.

[202] O’Callaghan E.O., Sanchez J.M., Rabaglino M.B., McDonald M., Liu H., Spencer T.E., Fair S., Kenny D.A., Lonergan P. (2022). Influence of sire fertility status on conceptus-induced transcriptomic response of the bovine endometrium. Front. Cell Dev. Biol..

[203] Peixoto P.M., Bromfield J.J., Ribeiro E.S., Santos J.E.P., Thatcher W.W., Bisinotto R.S. (2023). Transcriptome changes associated with elongation of bovine conceptuses I: Differentially expressed transcripts in the conceptus on day 17 after insemination. J. Dairy Sci..

[204] Peixoto P.M., Bromfield J.J., Ribeiro E.S., Santos J.E.P., Thatcher W.W., Bisinotto R.S. (2023). Transcriptome changes associated with elongation of bovine conceptuses II: Differentially expressed transcripts in the endometrium on day 17 after insemination. J. Dairy Sci..

[205] Biase F.H., Moorey S.E., Schnuelle J.G., Rodning S., Ortega M.S., Spencer T.E. (2023). Extensive rewiring of the gene regulatory interactions between in vitro-produced conceptuses and endometrium during attachment. Proc. Natl. Acad. Sci. Nexus.

